# Lateral interactions govern self-assembly of the bacterial biofilm matrix protein BslA

**DOI:** 10.1073/pnas.2312022120

**Published:** 2023-10-30

**Authors:** Sofia Arnaouteli, Natalie C Bamford, Giovanni B Brandani, Ryan J Morris, Marieke Schor, Jamie T Carrington, Laura Hobley, Daan M F van Aalten, Nicola R Stanley-Wall, Cait E MacPhee

**Affiliations:** 1 Division of Molecular Microbiology, School of Life Sciences, University of Dundee, Dundee, DD5 4EH, UK; 2 Department of Biophysics, Graduate School of Science, Kyoto University, Japan; 3 National Biofilms Innovation Centre, School of Physics & Astronomy, University of Edinburgh, EH9 3FD Edinburgh, UK; 4 UB Education, Content & Support, Maastricht University; 5 Sir William Dunn School of Pathology, University of Oxford, South Parks Road, Oxford, OX1 3RE, UK; 6 School of Biosciences, University of Nottingham, Nottingham, UK; 7 Department of Molecular Biology and Genetics, University of Aarhus, Aarhus 8000, Denmark

**Keywords:** Biofilm matrix, protein assemblies, X-ray crystallography, *Bacillus subtilis*, molecular dynamic simulations

## Abstract

The soil bacterium *Bacillus subtilis* is a model organism to investigate the formation of biofilms, the predominant form of microbial life. The secreted protein BslA self-assembles at the surface of the biofilm to give the *B. subtilis* biofilm its characteristic hydrophobicity. To understand the mechanism of BslA self-assembly at interfaces, here we built a molecular model based on the previous BslA crystal structure and the newly determined crystal structure of the BslA paralogue YweA. Our analysis revealed two conserved protein-protein interaction interfaces supporting BslA self-assembly into an infinite 2d lattice that fits previously determined transmission microscopy images. Molecular dynamics simulations and *in vitro* protein assays further support our model of BslA elastic film formation, while mutagenesis experiments highlight the importance of the identified interactions for biofilm structure. Based on this knowledge, YweA was engineered to form more stable elastic films and rescue biofilm structure in *bslA* deficient strains. These findings shed new light on protein film assembly and will inform the development of BslA technologies which range from surface coatings to emulsions in fast-moving consumer goods.

## Introduction

In the natural environment, bacteria can live in biofilms, communities of cells encased in a protective, extracellular polymer matrix consisting of eDNA, polysaccharides, and proteins (1). Biofilm matrix components have been studied in a plethora of bacterial species, where diverse functions have been uncovered (2, 3). Nonetheless how these components assemble within the matrix and interact with each other to form a mature biofilm and give rise to its emergent properties remains largely unknown for most species.

The biofilm matrix of the Gram-positive, soil-dwelling, bacterium *Bacillus subtilis* is predominantly comprised of exopolysaccharide and protein components (2). The major proteins include the fibres formed by the protein TasA (4, 5) with the aid of TapA (6, 7), and BslA (8), which is essential for giving *B. subtilis* pellicle and colony biofilms their characteristic wrinkled structure and high hydrophobicity (9). BslA is found at the external surface of the biofilm, where it forms a protective layer that has been described as a biofilm “raincoat” (10). X-ray crystallography revealed that BslA has an amphiphilic structure, with a three-stranded hydrophobic cap appended from a hydrophilic Ig-like domain (10). The hydrophobic amino acid side chains of the cap remain mostly buried when the monomeric protein is in aqueous solution but become exposed, via a conformational change, upon adsorption to an air/water or oil/water interface, increasing association strength (11, 12). BslA then self-assembles at the interface to form a highly ordered 2D lattice visible by transmission electron microscopy (TEM), and macroscopically observed by pendant drop tensiometry as an elastic protein film (11). Amino acid substitutions in the cap region to add hydrophilic groups decrease both the interfacial activity of the protein (12) and biofilm hydrophobicity (10).

The emergence of biofilm hydrophobicity also relies on the C-terminal region of BslA, which contains two cysteine residues (the “CxC” motif) that can cause dimerization and tetramerization of the protein (13). However, amino acid substitutions in this region, unlike those in the cap, do not abolish the ability of BslA to self-assemble into a strong elastic film at interfaces, nor do they extensively alter the typical wrinkled structure of the biofilm (2). Insight to BslA self-assembly has emerged from analysis of natural variants. In addition to BslA, some *Bacillus* species also encode a BslA paralogue, YweA. In *B. subtilis*, YweA shares 67% sequence similarity with BslA (14). *B. subtilis* YweA has conserved hydrophobic cap regions but lacks the C-terminal extension with the CxC motif necessary for dimerization and oligomerization (13, 14). While YweA can form ordered interfacial films *in vitro*, these are unstable, and heterologous expression in a *bslA* deficient strain does not rescue biofilm structure or hydrophobicity (13). The addition of the BslA CxC motif to *B. subtilis* YweA (referred to as YweA^CxC^) induces colony hydrophobicity and biofilm wrinkling when expressed in a *bslA* deficient strain. However, whereas the YweA^CxC^ variant increased biofilm structure, the removal or mutation of the CxC motif in BslA had little effect on biofilm structure or *in vitro* film stability (13). These results suggest key differences between the paralogues - in addition to those within the cap region and CxC motif that have previously been characterised – contribute to stronger film formation and biofilm structuring by the BslA family. These differences may be in the lateral protein-protein interactions that underpin film formation and may also contribute to the formation of the wrinkled biofilm architecture.

In this work, we set out to understand the molecular mechanism of BslA lateral self-assembly and its relevance for biofilm architecture. In addition to being an open question of fundamental biological interest, our understanding of BslA 2D ordering has relevance for the broader field of protein self-assembly and potential technological applications (15). Both BslA and YweA proteins form ordered lattices as determined by TEM, but only the molecular structure of BslA has previously been published. Here, we present a model of the 2D lattice based on the X-ray crystal structure of BslA and the newly determined crystal structure of YweA. The crystal structures suggest two distinct protein-protein interaction interfaces. We identify the key interactions holding these interfaces together and test these through a combined simulation and experimental approach. Finally, we show how self-assembly of the protein film positively correlates with colony biofilm structure and sporulation of the resident cells.

## Results

### YweA is structurally homologous to BslA

**Figure 1:**
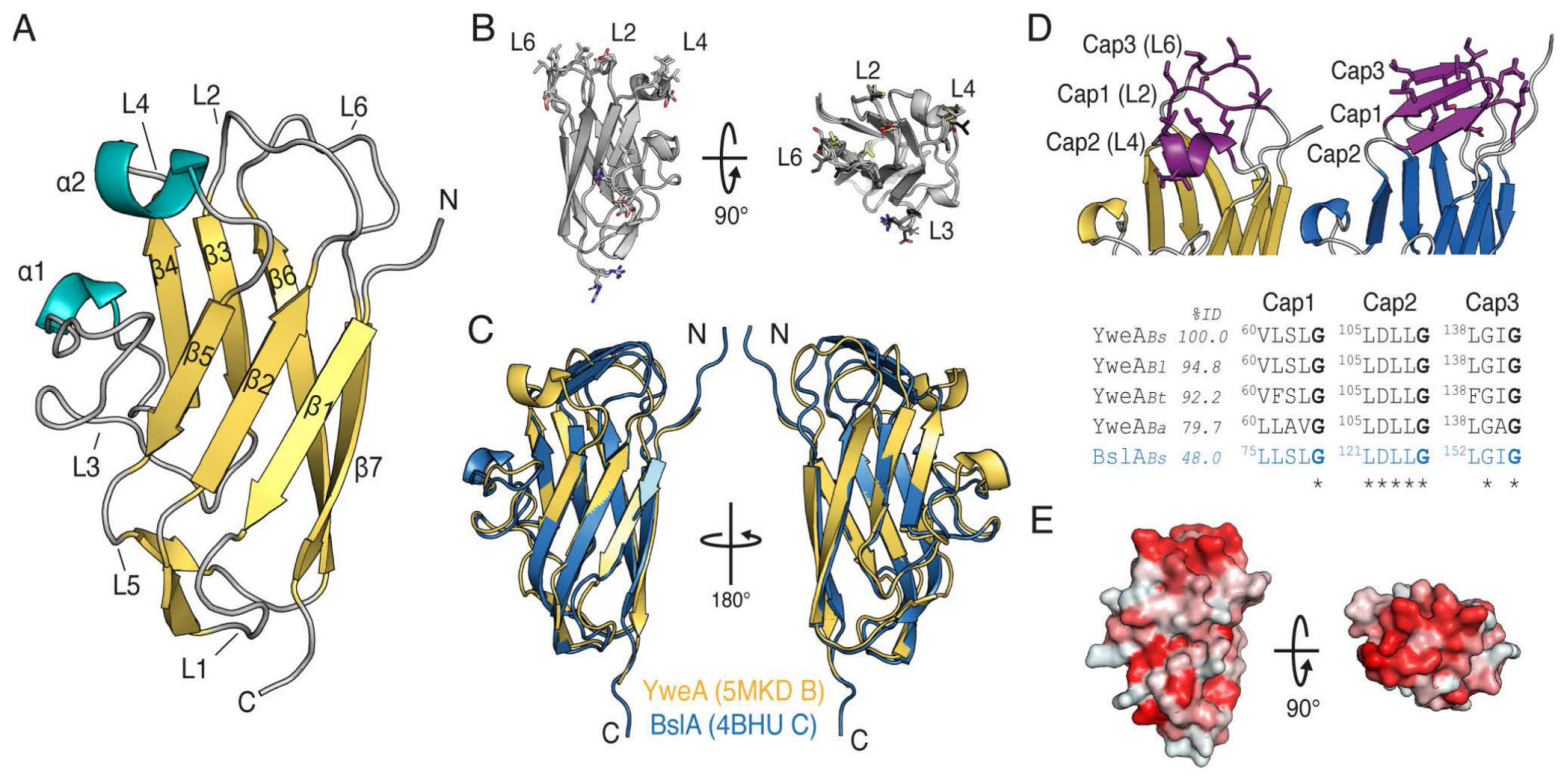
YweA adopts a β-sandwich fold similar to BslA. ***A.*** Cartoon representation of YweA (PDB 5MKD, chain B) coloured by secondary structure. The β-strands are yellow, α-helices teal, and loop regions in grey. The N- and C-termini are labelled (“N” and “C” respectively) along with loops (L), β-strands (β), and α-helices (α) which are numbered by when they occur within the primary amino acid sequence. ***B***. Structural alignment of the four monomers of the asymmetric unit from the side of the sandwich and from the top showing the variation in loops 2, 3, 4, and 6 between monomers. Sidechains of the poorest aligning residues are displayed (chain A: light grey, B: yellow, C: medium grey, D: dark grey). ***C***. Superposition of BslA (PDB 4BHU, chain C, blue) onto YweA (PBD 5MKD, chain B, yellow) shows the structural similarity between the core Ig-fold of the two paralogues. ***D***. Comparison of loops L2, L4, and L6, of YweA to the cap region of BslA shows similar exposed hydrophobic residues but differences in secondary structure. The cap residues are coloured purple whereas the other secondary structure elements are coloured yellow for YweA and blue for BslA. Alignment of the three cap regions of YweA with BslA shows conservation of hydrophobic residues. Cap regions are numbered based on their location in the primary amino acid sequence (Cap1 to 3). Sequence identity (%ID) is listed as compared to YweA from *B. subtilis* (YweA_*Bs*_) calculated over the full sequence alignment. Sequences of YweA homologues from *Bacillus licheniformis* (*Bl*), *Bacillus tequilensis* (*Bt)*, and *Bacillus amyloliqufaciens* (*Ba*) as well as BslA from *B. subtilis* (blue) were included. The amino acid number is listed for the first residue in each cap region. ***E***. Surface representation of YweA showing surface hydrophobicity coloured from least hydrophobic (white) to most hydrophobic (red) based on Eisenburg hydrophobicity scale.

To determine whether YweA could aid our understanding of stable interfacial film formation by BslA, we first sought to obtain the structure of the BslA paralogue for comparison. The atomic structure of YweA from *B. subtilis* (YweA residues 31-155, excluding the predicted signal peptide, referred to as YweA herein) was determined using X-ray crystallography to a resolution of 2.51 Å (Table S1). YweA crystallised in space group *C222* with four monomers (chains A to D) in the asymmetric unit. Each monomer adopts a seven-stranded β-sandwich resembling an immunoglobulin (Ig) domain (Figure 1A), with an additional two-stranded sheet at one end of the fold and two small helices. The conformations of the four YweA structures in the asymmetric unit are similar, with pairwise root-mean-square deviations (RSMD) ranging between 0.4-0.5 Å over all α-carbons, and 1.0-1.3 Å over all atoms (Figure 1B).

Submission of YweA to the protein structure comparison server Dali (16) revealed the most similar deposited structure was BslA, from *B. subtilis* (PDB 4BHU) (10). The structural similarity of YweA and BslA was expected as these proteins are paralogues with a sequence identity of 48% (clustalW (16)). Structural alignment of BslA to YweA revealed high overlap between the core Ig-fold with root-mean-square deviations (RMSD) of 1.2Å over 115 of 124 α-carbons (Figure 1C). Of the region aligned, the sequence identity was 50.4%. Structural differences were found at the N- and C-termini where BslA has extensions that are lacking in YweA (Supplemental Figure 1). The other marked structural differences are in the loops (Ls) 2, 4, and 6 of YweA which correspond to the “cap” region of BslA (Figure 1CD). In BslA, these residues are highly conserved and have been found to be structurally flexible (14). In the BslA crystal structure, the cap forms β-sheets in eight of the ten monomers of the asymmetric unit, with the other two monomers having unstructured caps(10). For YweA, all monomers lack β-strand formation and instead have two loops without secondary structure and one helix. The YweA cap region is the site of the highest surface hydrophobicity (Figure 1E), and the largest structural variability between the four monomer structures (Figure 1B), suggesting structural flexibility. YweA, like BslA, can undergo structural changes at an oil/water interface wherein YweA gains β-sheet structure (14). The inferred flexibility of the cap loops in YweA, and their high sequence identity to BslA (Figure 1D), suggests that this region could transition into a β-sheet as seen in BslA and in previous studies (10). Thus, it is possible that YweA orients at the interface similarly to BslA and the lateral interactions would then occur between equivalent protein surfaces. The structural homology of YweA to BslA, and the detection of a similar flexible hydrophobic cap, encouraged us to consider the different packing arrangements within crystals to explore whether the YweA structural data can be used as a proxy to determine the lateral interactions between BslA monomers.

### YweA and BslA crystal structures reveal two distinct dimer interfaces

We investigated whether the crystal structures of BslA and YweA could provide information on the protein-protein interactions that might occur *in vivo*. Interactions between protein units *in crystallo*, either within and/or between asymmetric units, could reflect similar interactions at an interface. We looked at the interactions between monomers in the BslA and YweA asymmetric units to establish if any interactions were present that allowed for the cap regions to face the same direction, as would be expected at a hydrophobic/hydrophilic interface.

**Figure 2:**
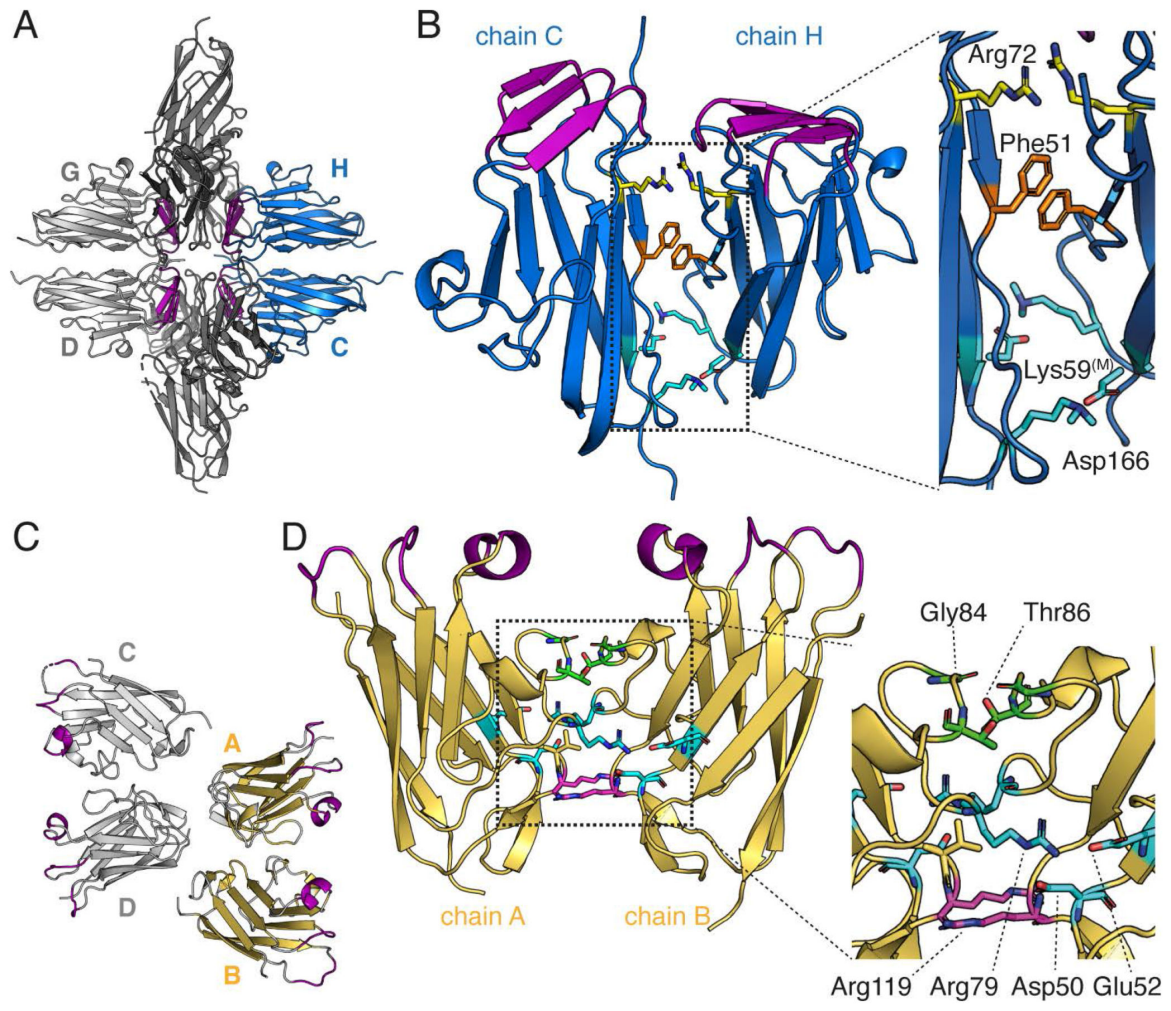
The crystal structures of BslA and YweA cap-aligned dimers. ***A*,** Cartoon representation of the BslA asymmetric unit that consists of 10 monomers. Two dimers are present that have cap regions (purple) aligned with their partners (H with C [blue] and G with D [light grey]). ***B*,** Cartoon representation of dimer1 (chain C and H) of BslA PDB 4BHU. BslA is displayed in blue with the cap region in purple. Residues that promote interaction at the interface are displayed with sidechains showing the stacking of Arg72 (yellow), π-π stacking of Phe51 (orange), and positioning of the methylated Lys59 (Lys59^(M)^) within hydrogen bonding distance of Asp166 (cyan). ***C*,** Cartoon representation of the YweA asymmetric unit that consists of four monomers. Two dimers are present that have cap regions (purple) aligned with their partners (A with B [yellow] and C with D [light grey]). ***D*,** Cartoon representation of dimer2 containing chain A and B of YweA PDB 5MKD. YweA is displayed in yellow with the cap region in purple. Residues that interact at the interface are shown in stick representation and coloured by interacting groups. The inset shows this zoomed in with residues labelled.

BslA crystallised in a decameric micelle configuration (Figure 2A). The micelle constitutes the asymmetric unit of the crystal and contains ten monomer structures labelled as chains A through J in PDB 4BHU. This packing centres around the cap regions of the monomers, protecting them from solvent exposure. From the X-ray structure of BslA, two equivalent dimers, corresponding to chains C and H and chains D and G, have aligned cap regions (Figure 2B). The C/H dimer will be representative of this set and will be referred to as “dimer1” hereafter (Figure 2B). The YweA crystal asymmetric unit can be divided into two equivalent dimers (chains A and B are equivalent to chains C and D) with cap regions that align to create one interface each (Figure 2C and 2D). We refer to this YweA dimer as “dimer2”. Dimer1 and dimer2 represent two different orientations of two monomers and thus constitute unique dimer interactions.

To investigate the dimer interfaces within the crystal, the structures of both BslA and YweA were submitted to the PISA web tool (17). The dimer1 interface was found to have 16 buried residues from each monomer with an average buried surface area of 472 Å^2^ per monomer. Dimer2 consisted of 17 residues from chain A and 16 from chain B with an average buried surface area of 603 Å^2^. In isolation, these buried areas are at the low end of the 600-1600 Å^2^ range of homodimer structures (18), although it should be noted that YweA is a relatively small protein. In addition to the buried hydrophobic residues that may contribute to dimer assembly, there are stacking and electrostatic interactions between monomer sidechains in each dimer. Sidechain interactions at the BslA dimer1 interface include the stacking of Phe51 to Phe51 and Arg72 to Arg72 (Figure 2B inset). There is also the potential for a symmetrical set of salt bridges between Lys59 and Asp166, but this is not seen in the structure as Lys59 was methylated for crystallisation (Figure 2B). The dimer2 interface of YweA includes hydrogen bonding between Asp50 and Glu52 of chain A with Arg79 chain B, and vice versa (Figure 2D inset). Arg119 also hydrogen bonds with the backbone amide of Arg119 on the other monomer. The backbones of loop 3 (L3) of both monomers also hydrogen bond each other (Thr86 amide nitrogen to Gly84 oxygen). It is possible that the loop shifts upon dimerization as slight conformational differences are seen between the monomers of the crystal packing unit (Figure 1B). The residues involved in both dimer interfaces are highly conserved within each paralogue family (Supplemental Figure 1). Despite the low buried surface area, the conservation and the alignment of the hydrophobic caps supports that these dimer orientations may constitute two unique lattice-forming interactions.

### Dimer interfaces suggest a BslA lattice

**Figure 3:**
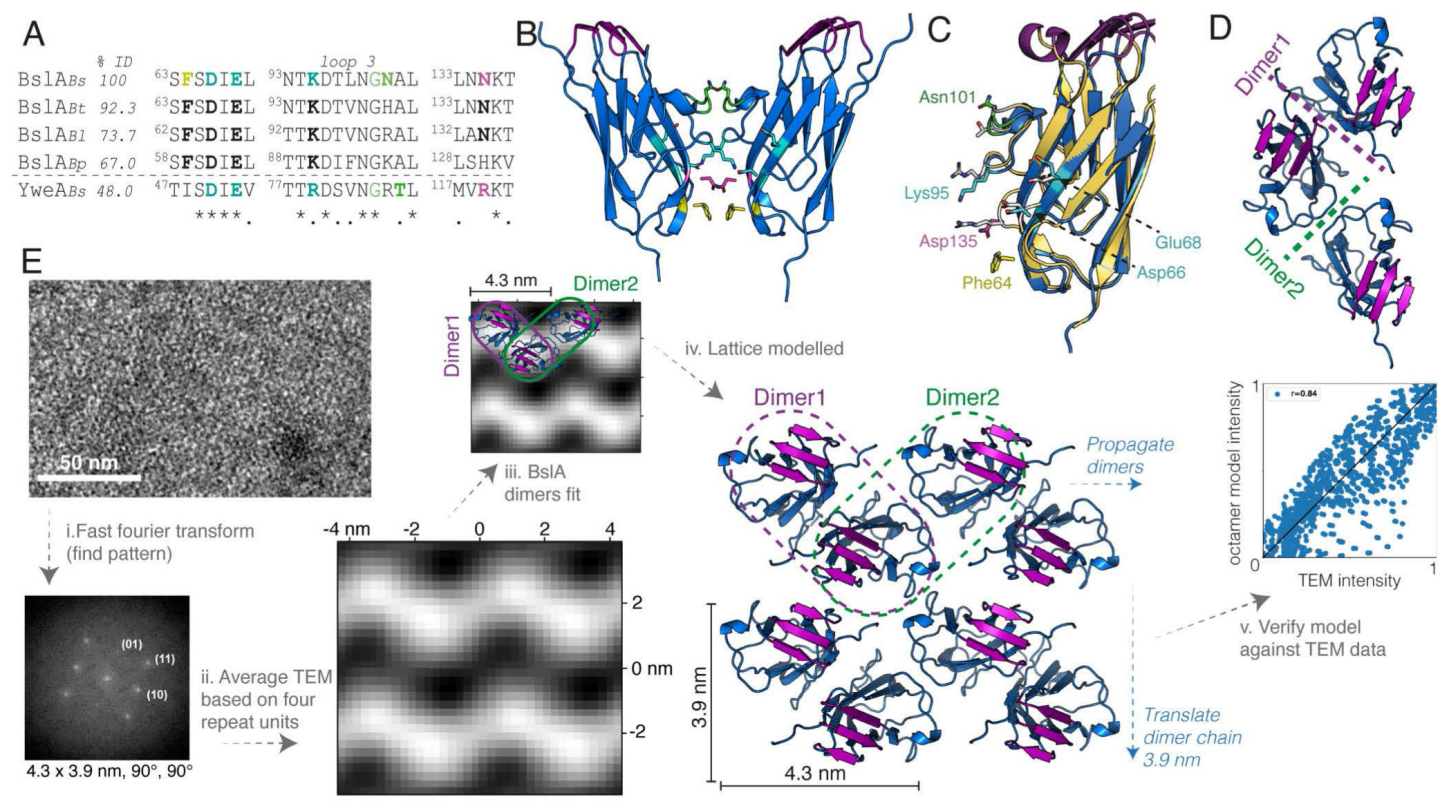
Construction of a model BslA lattice. ***A***. Sequence alignment of BslA homologues (*Bs*: *B. subtilis*, *Bt: B tequilensis*, *Bl:B. licheniformis*, and *Bp: B. pumilis*) with YweA from *B. subtilis* shows conservation of some of the dimer2 interface residues between paralogues. Residues that are shown in panel *B* are in bold and coloured accordingly. Sequence identity is listed based on alignment over the entire sequence as compared with BslA*_B_S__*. The first residue of each region is numbered based on its location in the primary sequence. ***B*.** and ***C*.** Structural model of BslA dimer2 created from the alignment of BslA monomers onto the YweA dimer2. Cap regions are shown in purple. YweA is in yellow and BslA is in blue. Residues that could interact at the BslA dimer interface are coloured and labelled with the YweA residues in grey (panel *C* only). The amino acid labelling refers to BslA. ***D*.** A model trimer created from aligning a monomer of dimer1 with a monomer of dimer2. The dimer interfaces are shown with dashed lines and labelled. ***E*.** A 2-dimensional BslA lattice was produced based on the crystallographic dimers described herein and the TEM data from Bromley *et al*. (11) The first step (i.) was the fast Fourier transform (FFT) to determine the repeat unit of the ordered BslA lattice (x = 4.3, y = 3.9, α=β= 90°). Step (ii.) was the averaging of the TEM image to see four repeat units (octamer) where brighter pixels represent higher electron density. The modelled trimer (dimer1circled in purple and dimer2 circled in green) is congruent with the TEM pattern (iii.). A lattice can be constructed (iv.) by the structural alignment of monomers from the crystallographic dimers which creates a chain across the page. Translation of the propagated dimers by 3.9 nm (y-direction) leads to a hypothetical 2D lattice that could extend infinitely. The repeating units measure 4.3 nm by 3.9 nm in agreement with the FFT. The lattice is shown with the caps (purple) all facing out of the page. Verification of the model (v.) was done by comparison of the electron density of the model (converted to hypothetical TEM intensity) with the intensity of the TEM image over each pixel shows a good correlation.

Film formation at an interface likely involves multiple lateral interactions. Having identified two potential dimer interfaces, we next explored whether BslA might form an analogous dimer2 interface to that formed by YweA. We initially looked at whether the interacting amino acids were conserved between paralogues (Figure 3A), in addition to the observed conservation *within* paralogues. We produced a potential BslA dimer2 *in silico* by aligning two BslA monomer structures to the YweA dimer2 structure (Figure 3B). This model of the dimer only presents a guide to possible interactions, as formation of the protein-protein interface would probably lead to small structural rearrangements. We uncovered that the YweA dimer2 salt bridge is partially conserved, with Asp50 and Glu52 conserved in BslA (Asp66 and Glu68), whereas Arg79 is replaced in BslA by lysine in the primary sequence (Lys95) (Figure 3C). All three residues (Asp66, Glu68, and Lys95) are conserved between BslA homologues (Figure 3A and Supplemental Figure 1). In the model BslA dimer2 interface, Lys95 is positioned such that it could create a salt bridge with Asp66. Glu68 is positioned further away than the equivalent YweA residue (Figure 3C). Lys and Glu have flexible sidechains so interaction *in vivo* may be possible. YweA Arg119 is not found in BslA, instead, a well-conserved asparagine (Asn135) is found at this location. In the aligned structure, it appears that Asn135 could create hydrogen bonding interactions with the Asn135 of the opposite monomer (Figure 3B). Additionally, BslA Phe64 is conserved within the BslA family and could participate in π-π stacking during dimerization (Figure 3B and Supplemental Figure 1). L3 of YweA aligns well with the reciprocal loop of BslA, suggesting that the BslA backbone could hydrogen bond between monomers as seen in YweA. BslA Asn101 aligns structurally with YweA Thr86 of L3 (Figure 3C).

Based on the sequence conservation within BslA homologues, and the structural alignment, it seems possible that BslA could multimerise using a similar interface to YweA dimer2. Combining the dimer1 and newly modelled BslA dimer2 was done by aligning one monomer of each to create a trimer (Figure 3D). This trimer had no structural clashes, suggesting that both interactions could occur at the same time at an interface. Next, to determine whether the model trimer could be relevant to BslA lattice formation we compared it to the published TEM data (Figure 3E)(11). Fast Fourier transform (FFT) of the TEM image (Figure 3Ei) determined a rectangular lattice unit cell of 4.3 nm by 3.9 nm(11). Figure 3Eii shows the TEM average density of the unit cell repeated twice along each dimension, highlighting eight bright patches creating two rough zigzags. We found that the model trimer fits the shape of the low-resolution image (one “zig”, Figure 3Eiii), with each protein corresponding to a patch within the unit cell, suggesting that the unit cell should be made of exactly a BslA dimer.

The two dimers constitute protein interactions that can be extended infinitely in one dimension with a repeat unit of approximately 4.3 nm, consistent with the TEM data. Knowing that the lattice has a rectangular unit cell and guided by the low-resolution TEM image of an octamer (Figure 3Eii), we can model a lattice in which the propagated dimer zigzag is shifted 3.9 nm perpendicularly to produce a BslA lattice that can be extended infinitely in two dimensions (Figure 3Eiv). A comparison of the 2d densities of the modelled lattice octamer and the TEM 2x2 unit cell patch shows a high Pearson correlation coefficient of r ~ 0.84 (*p*-value ~ 10^-300^, Figure 3Ev), thus supporting our model. Furthermore, we note that the TEM lattice has P2 symmetry, and our modelled BslA dimers indeed display the two-fold symmetry that is required to generate such a lattice.

### Both BslA dimers are stable in molecular dynamics simulations

**Figure 4:**
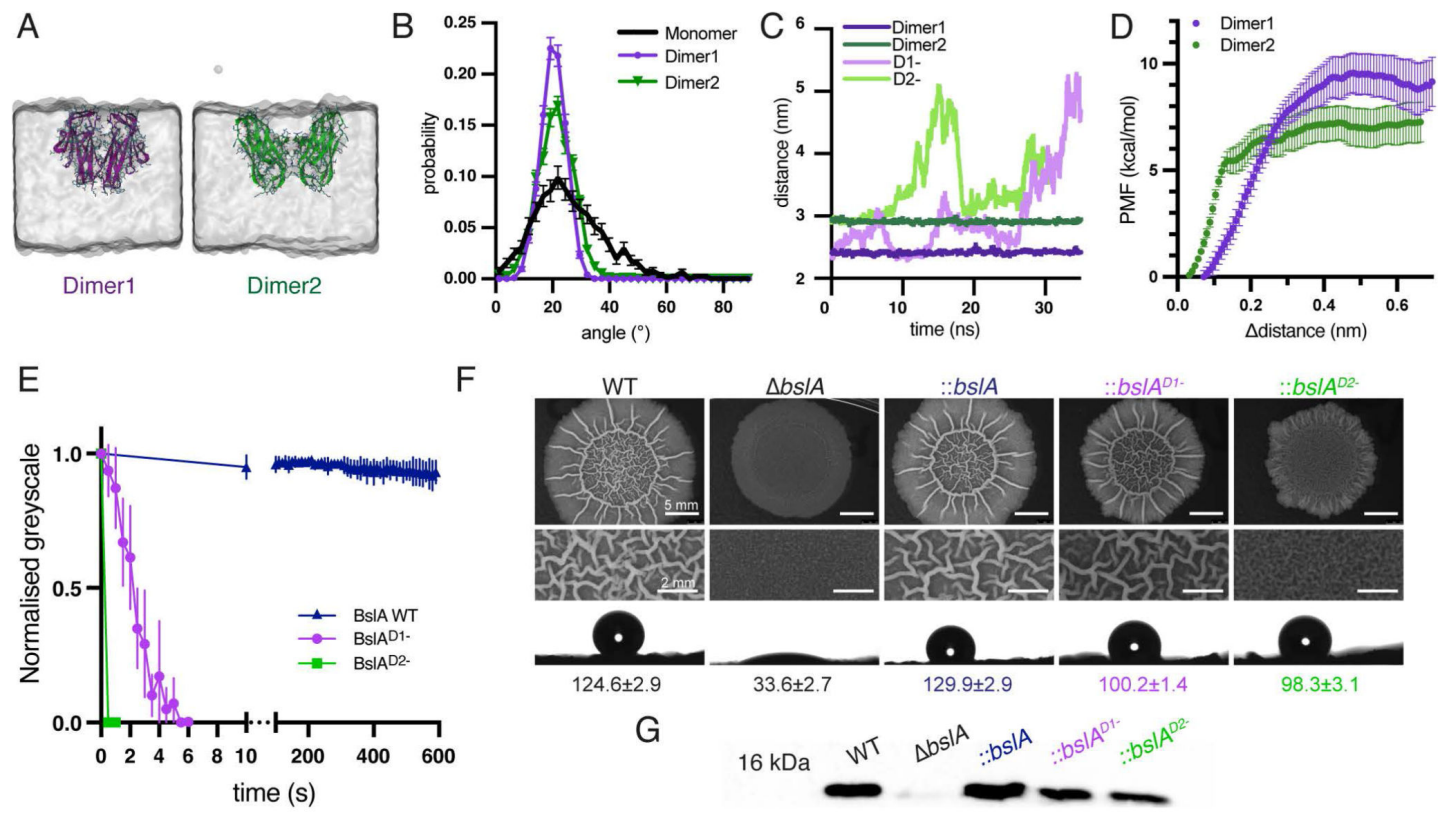
Molecular dynamic simulations support dimer orientations. ***A*.** Snapshots of the MD simulation at equilibrium (t = 100 ns) for BslA dimer1 (purple) and dimer2 (green) in an aqueous box with an air interface. ***B*.** Orientation of BslA monomer (black), dimer1 (purple circles), and dimer2 (green triangles) in relation to the normal of the interface as determined from equilibrium MD simulations. ***C*.** Measure of the distance (nm) between BslA monomers during non-biased equilibrium MD simulations for the first 35 ns, for Dimer1 (dark purple), Dimer2 (dark green), BslA^D1-^ (light purple), and BslA^D2-^ (light green). ***D*.** PMF is indicative of the binding affinities of the dimer1 interface (purple) and the dimer2 interface (green). The force in kcal/mol was graphed against the change in distance (nm) from the equilibrium position of the pulled monomer. The bars represent the errors on our PMF estimate. ***E*.** wrinkle relaxation graphed by the change in normalised greyscale of pendent drop wrinkles over time (s) for BslA WT (blue), BslA^D1-^ (light purple), and BslA^D2-^ (light green). Error bars represent standard deviation. ***F*.** Representative colony biofilms and sessile drop images. Hydrophobicity measures are listed representing the angle of the edge of the droplet to the base n = 3, errors represent the SEM. A contact angle of greater than 90° is indicative of a non-wetting/hydrophobic surface. ***G*.** Immunoblot of the matrix localised BslA variant from the strains analysed in *F*.

To investigate the validity of the lattice model described above we turned to simulations. Previous coarse-grain molecular dynamics (MD) simulations of BslA determined that the hydrophobic cap is important for protein orientation at an interface (12). We reasoned that monomers may reach the interface and then associate laterally to make a lattice (11). Considering this, we sought to determine if the orientation of the dimers was congruent with the equilibrium orientation of monomers at the interface. All-atom equilibrium MD simulations were performed in which the BslA monomer, crystallographic dimer1, or modelled dimer2 were absorbed to an air-water interface (Figure 4A). These simulations revealed that the angle that the dimers make to the interface sits within the distribution of the equilibrium orientations of the monomers (Figure 4B). Additionally, the peaks of each orientational distribution (monomer, dimer1, and dimer2) align well (Figure 4B). The distribution of dimer orientations was less broad than the monomer, suggesting dimerization constrains the low-energy landscape (Figure 4B). Thus, monomers could dimerize after reaching an interface without having to significantly change orientation to do so. With this setup, we were also able to investigate the stability of the dimers by determining whether the distance between the units changed over the course of the simulation. Spontaneous separation/dissociation of the units would suggest low stability. The simulations showed no separation events over the timescale of the simulation (100 ns) (Figure 4C, showing the first ~35 ns of MD). These equilibrium MD simulations also offer the opportunity to test the presence of specific lateral interactions that could not be observed directly in the crystal structures. For dimer1, the simulations confirm the role of a salt bridge between Lys59 and Asp166, which is formed in 86% of the conformations sampled by MD (based on a 4 Å cutoff distance between the carboxylate carbon and the ammonium nitrogen). For dimer2, the simulations confirm the presence of the loop-loop backbone hydrogen bonds, the salt bridge between Lys95 and Asp66 (formed 99% of the time), and occasional stacking of Phe64 side chains; however, we did not find stable electrostatic interactions between Asp135 side chains, whose orientations are not optimal for hydrogen bonding, nor salt bridges between Lys95 and Glu68, which remain too far to interact.

Next, to quantify the energetics of the protein-protein interaction within the dimers, the potential of mean force (PMF) along the distance between the monomers was calculated for each of the BslA dimers using steered MD simulations (Figure 4D). These simulations revealed that dimer1 had a stronger protein-protein interaction, with a standard free-energy of binding (△*G*_bind_) of 11 ± 1 kcal/mol, compared to 8 ± 1 kcal/mol for dimer2 (see Materials and Methods section for the calculation of △*G*_bind_) (19). These values are smaller than the binding free energy of the barnase-barstar system, with △*G*_bind_ 19 kcal/mol (20), but they are within the range of values expected for typical protein-protein complexes, as estimated in a recent MD investigation (△*G*_bind_ ~6-23 kcal/mol) (19). We note however that binding free energies in 2 and 3 dimensions are not directly comparable because of the different degrees of freedom, and that in our case the energies from distinct interfaces would be additive within the context of the regular lattice.

### Mutation of BslA dimer interfaces affects function

To assess the relevance of the two putative dimer interfaces with respect to BslA film formation, we designed variants that would disrupt the two interfaces. To disrupt the dimer1 interface, Phe51 was replaced with alanine to remove the π-π stacking interaction and Asp166 was replaced with lysine to disrupt the two symmetric salt bridges and lead to repulsion. The variant, BslA Phe51Ala Asp166Lys, will be referred to as BslA^D1-^. To disrupt the protein interface of dimer2, Asn101 was replaced with aspartic acid to prevent the possibility of sidechain-sidechain hydrogen bonding and induce repulsion between L3 of the two monomers. Asp66 was also replaced with lysine to disrupt the putative symmetric salt bridges. The resultant variant form, BslA Asp66Lys Asn101Asp, is henceforth termed BslA^D2-^. The validity of our amino acid substitution selection was confirmed using the equilibrium MD simulations. In contrast to the wild-type counterparts, the variant dimers were found to spontaneously separate when absorbed onto an interface (Figure 4C).

We next determined if the dimer1 and dimer2 interfaces were important for BslA function *in vitro* and *in vivo*. Recombinant BslA representing the secreted domain (residues 42-181, referred to as BslA herein) and the two BslA dimer interface variant forms, BslA^D1-^ and BslA^D2-^, were produced in *E. coli* using a method previously established (10). The amino acid substitutions did not have any significant impact on protein structure as determined by circular dichroism (CD) spectroscopy of the variant proteins (Supplemental Figure 2). Next, the impact of amino acid substitutions on *in vitro* protein film formation was assayed using pendant drop wrinkle relaxation experiments. Pendant drop experiments were performed using oil as the hydrophobic phase (10). Wrinkles in the film formed by wild-type BslA do not significantly relax over the assay period of ten minutes (Figure 4E). By contrast, wrinkles in the film formed by the BslA^D1-^ protein relaxed within 6 s, and for BslA^D2-^ the wrinkles disappeared almost immediately (Figure 4E). These findings demonstrate that the residues substituted are required for interfacial film stability *in vitro*.

To examine the role of the residues required for dimer1 and dimer2 formation *in vivo*, the ability of the BslA dimer variants to recover hydrophobicity and colony biofilm structure when produced within a *bslA* deletion strain was tested (see Table S2). Figure 4F demonstrates that expression of the genes encoding either BslA^D1-^ or BslA^D2-^ successfully recovered hydrophobicity in the *bslA* mutant strain. However, while production of BslA^D1-^ in the *bslA* background (NRS5526) fully recovered colony biofilm architecture to that displayed by the wild-type strain (NRS2299), the colony biofilm formed by the strain producing BslA^D2-^ (NRS5524) lacked the highly wrinkled structure associated with a mature wild-type biofilm (Figure 4F). The presence of each variant in the biofilm colonies was verified via immunoblot analysis using an anti-BslA antibody against the corresponding protein extracts (Figure 4G). Collectively, these results indicate that the residues required for dimer1 and dimer2 interfaces are required for stable film formation *in vitro* and that disruption of the dimer2 interface interactions has an impact on biofilm structure *in vivo*.

### Engineering YweA surface residues increases film strength

**Figure 5:**
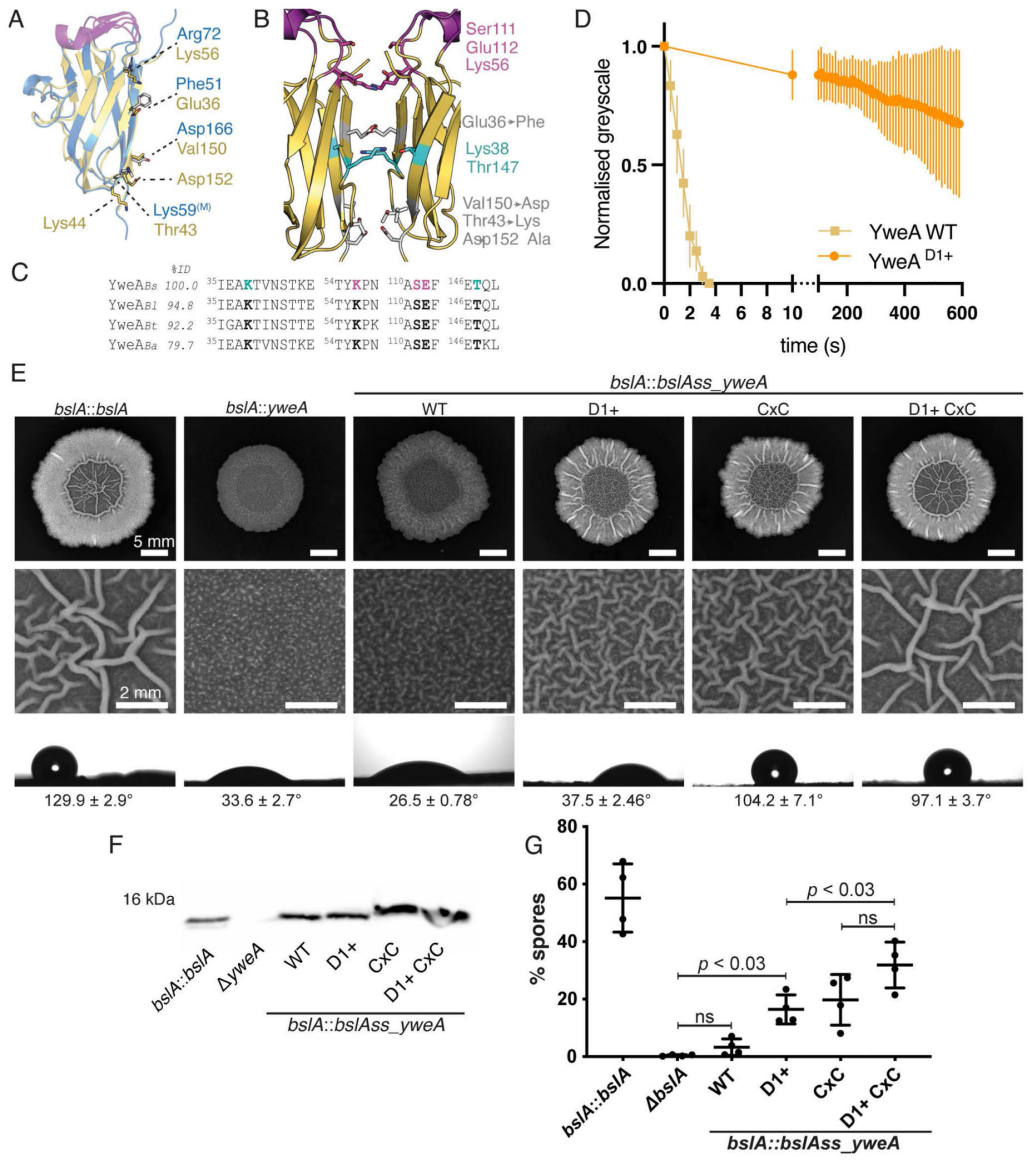
Engineering of YweA dimer1 interface increases film stability. ***A.*** Alignment of YweA (yellow) and BslA (blue) shows that the BslA dimer1 residues (grey, labelled in blue) are not conserved. Cap regions are coloured in purple for orientation. Aligned YweA residues and amino acid labels are shown in yellow. ***B*.** The model of the YweA dimer1 interface shows a possible hydrogen bonding network. The residues of note are pink and cyan. Residues in grey are those mutated to create the BslA-like dimer1 interface on YweA. ***C*.** Sequence alignment of YweA homologues (*Bs*: *B. subtilis*, *Bt: B tequilensis*, *Bl: B. licheniformis*, and *Ba: B. amyloliqufaciens*) shows conservation of the dimer1 interface residues. Residues that are shown as sticks in panel B are in bold and coloured accordingly. Sequence identity is listed based on alignment over the entire sequence as compared with YweA_*Bs*_. The first residue of each region is numbered based on its location in the primary sequence. ***D*.** YweA film relaxation after droplet compression (graphed by the normalised grey value) over time. YweA WT is graphed in yellow (squares) and the YweA^D1+^ mutant is in orange (circles). ***E*.** Representative colony biofilm images as well as zoomed-in in centre of each colony with hydrophobicity measures listed below. A contact angle of greater than 90° is indicative of a non-wetting/hydrophobic surface. ***F*.** α-YweA immunoblot of matrix proteins from the biofilms including a *yweA* deficient sample (NRS2405) as a negative control. ***G*.** Quantification of heat-resistant spores (sporulation) for each strain when grown as a colony biofilm plotted as the mean value over four biological replicates. Error bars are the standard deviation and statistics are shown as calculated from a one-way ANOVA with Šídák’s multiple comparisons test in GraphPad Prism 9.

Having been able to weaken BslA film formation by manipulating the interactions at the dimer interfaces, we next explored if the unstable film formed by purified recombinant YweA could be engineered to adopt the stable BslA film-forming properties. For the dimer1 interface (derived from the crystal packing of BslA), alignment of YweA onto BslA reveals that the residues integral to the BslA dimer1 interface are not conserved between the paralogues. In the place of the π-π stacking Phe51, YweA has Glu36 (Figure 5A). BslA Arg72 aligns with YweA Lys56 which also lacks π-π stacking ability, although it retains the positive charge. Although the sequence alignment suggests equivalent residues in YweA (Lys44 and Asp152) to the dimer1 salt bridge between Lys59 and Asp166 in BslA, the structure shows these residues are not positioned to interact (Figure 5B). Structurally BslA Lys59 and Asp166 align with YweA Thr43 and Val150, respectively. It is possible that YweA still shares this lattice interface as there are other interactions present. The YweA dimer1 model suggests that Lys56 could bind Ser111 and/or Glu112 creating a symmetric hydrogen bond/salt bridge (Figure 5B). This conclusion is based on the high flexibility of the lysine sidechain and that these residues are well conserved in YweA homologues (Figure 5C). There is also a potential hydrogen bond between Lys38 and Thr147, which are also conserved residues within the YweA group (Figure 5B and 5C). These two sites suggest the possibility of a shared dimer1 interface but with lowered affinity compared to that of BslA. We therefore designed a YweA variant, named YweA^D1+^, with BslA-like dimer1 residues. The variant included Glu36Phe, Thr43Lys, Val150Asp, and Asp152Ala, which would create one π-π stacking interaction and one new salt bridge as well as remove the possible repulsion that could occur between symmetric Asp152 sidechains (Figure 5B).

We investigated whether YweA^D1+^ could form a more stable BslA-like film *in vitro*. YweA WT and YweA^D1+^ proteins were produced recombinantly in *E. coli* and determined to have no significant secondary structure differences by circular dichroism (CD) (Supplemental Figure 2). The wrinkle relaxation assay (Figure 5D) shows that wrinkles in the film formed by YweA^D1+^ relax significantly slower than those in a film of YweA WT protein, with some wrinkles that did not relax during the experimental time frame. These results confirm the importance of the amino acid contacts we identified in stabilising film formation.

We next assessed whether the engineered dimer interface variant, YweA^D1+^, could mimic the function of BslA *in vivo*. Using the *bslA* deletion strain as the parental strain, *yweA* variants were generated such that expression was under the control of an IPTG inducible promoter and secretion was directed through the Sec-system driven by the BslA signal sequence. After construction of the strains, they were evaluated for their ability to form mature, hydrophobic, structured colony biofilms (Figure 5E) and the presence of each YweA variant was verified via immunoblot analysis using an α-YweA antibody against the corresponding protein extracts (Figure 5F). We used the level of sporulation as a proxy for the degree of biofilm maturation (21–23).

As anticipated, genetic complementation of the △*bslA* strain with the *yweA* gene *bslA* signal sequence chimaera (*bslAss*_*yweA*, NRS5551) led to only a limited change in colony biofilm structure and no significant increase in sporulation (Figure 5G) (13). In contrast, when the YweA^D1+^ variant was produced within the *bslA* deletion strain (NRS5542), while there was no recovery of biofilm hydrophobicity, there was a recovery in the complexity of the colony biofilm architecture back towards that displayed by the “wild type” strain (NRS2299) (Figure 5E). Additionally, a significant increase in the percentage spores in the population was observed, with an average of 16.4 % compared to 0.4 % from the △*bslA* parent strain (*p* < 0.03), demonstrating maturation of the biofilm community (Figure 5G). Our findings with the engineered YweA^D1+^ variant show that increased film strength correlates with increased biofilm structure and maturation.

### Encoding multiple BslA features within yweA yields BslA-like function

Despite the recovery of biofilm architecture observed upon production of YweA^D1+^, we concluded that the YweA^D1+^ variant was not able to fully rescue the △*bslA* phenotype based on the colony biofilm morphology (Figure 5E). Therefore, we constructed another variant of YweA engineered to contain the BslA-like dimer1 interface that enhanced film stability but also added the BslA “CxC” region previously observed to be important for biofilm hydrophobicity, named here YweA^D1+ CxC^ (NRS5541). We assessed if these engineered changes fully recapitulated BslA-like function *in vivo*. As an additional control, we used a strain producing the YweA^CxC^ variant (NRS4834); which was able to rescue colony biofilm structure and sporulation to a similar level as YweA^D1+^, but as expected, additionally could reinstate biofilm hydrophobicity (Figure 5E). We found that the YweA variant possessing the combined BslA attributes (YweA^D1+ CxC^) complemented the △*bslA* strain (NRS5541) giving a full wild-type-like colony biofilm structure with large central wrinkles and full surface hydrophobicity (Figure 5E). Moreover, the colony biofilm showed signs of further maturation with spores forming in the population at a significantly higher level than YweA^D1+^ (*p* < 0.03, Figure 5G). Taken together our results demonstrate that the protein-protein interactions we identified in the crystal structure are relevant to BslA and YweA film formation and biofilm architecture and recapitulated the importance of the CxC motif for biofilm hydrophobicity.

## Discussion

During biofilm formation microbes produce and secrete a diverse mixture of molecules that create a protective and structured matrix for the community. The matrix molecules also contribute to robustness and the emergent properties associated with biofilm formation (3). *B. subtilis* is a well-studied model for biofilm formation with the main components of the matrix established in literature (2). One such component, the secreted protein BslA, has been studied in detail (9–11, 13, 14, 24). The ability of BslA to form protein films and stabilise emulsions is interesting from a translational perspective, and moreover, this trait is necessary for its role in *B. subtilis* biofilm formation, structure, and protection (9, 12, 25). Despite the interest in BslA film properties, little was known about how the films assemble and the molecular interactions underpinning these elastic assemblies. Herein we used a combination of structural, biophysical, and microbiological approaches to determine specific protein interactions and their impact on BslA function.

The crystal structures of BslA, and its paralogue YweA, revealed two unique protein-protein interfaces that allow BslA to form two-fold symmetric dimers with the same hydrophobic cap orientation. Based on these dimerization interfaces, we build a hypothetical molecular model of the BslA interfacial lattice. Our model is supported by multiple independent lines of experimental evidence: first, the model quantitatively fits into the average lattice unit cell determined from previously published TEM images of BslA films (11); molecular dynamics simulations confirm the stability of these BslA dimers at interfaces, including the one modelled from the YweA crystal structure; mutations at the key interfaces weaken the BslA elastic films *in vitro*, and, for one interface, also alter the morphology of the biofilm; finally, YweA mutations made to resemble BslA at one key interface contributes to rescuing the biofilm morphology in △*bslA* strains. Confirmation of the atomistic details of our molecular model would require additional structural characterisation using e.g., cryo-EM, however, collectively our data show that the identified molecular interactions form the basis of BslA self-assembly both *in vitro* and within the actual biofilm.

We note that the lattice model suggests an arrangement of BslA monomers with four interaction interfaces (each monomer binds four neighbours) (Figure 4), but from the available crystal structures, we could only characterize two of them in detail. Future work could be done to explore the other two interfaces and their impact on film strength, stability, and dynamics. The reason why these two interfaces do not appear in the available crystal structures could be due to their relative weakness compared to the other interfaces, although it could simply be due to unfavourable crystal packing or the crystallisation conditions which may weaken or strengthen ionic/non-ionic interactions. The identification of dimer1 interface from the BslA crystal structure, and its higher free energy of binding compared to BslA dimer2 from the MD simulations, supports that this interface could be the strongest of the four BslA protein-protein interactions.

**Figure 6:**
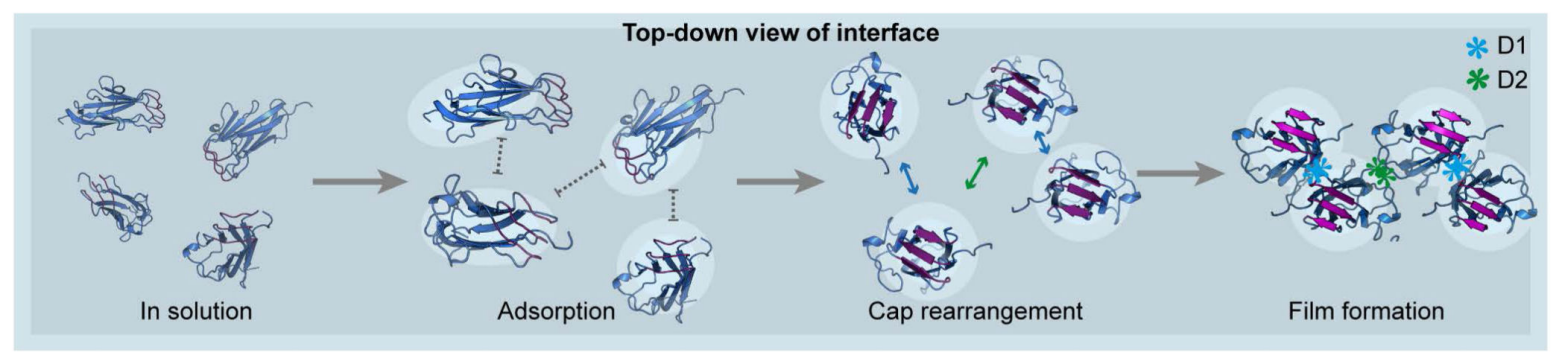
Model of BslA film formation. BslA exists in solution in a soluble “cap in” configuration (for simplicity BslA is only shown in its monomeric form but in solution can also exist as dimers and tetramers mediated via disulfide bonds through their CxC motifs, unrelated to the interfacial lateral interactions (13)). BslA absorbs onto an interface and undergoes a limited structural rearrangement into the “cap out” form (11), which exposes the cap hydrophobic residues and reorientates the protein (12), facilitating lateral self-assembly. The final cartoon illustrates the D1 and D2 lateral interactions that our experimental *in vitro* and *in vivo* data support as the molecular basis for interactions between monomers that hold the film together.

The MD simulations of the orientation of monomers and dimers at air/buffer interface revealed a good correlation. The dimers were more constrained compared to monomers, which makes sense as there is a greater hydrophobic surface created by the two exposed cap regions. The orientation of dimer1 and dimer2 were overlapping, supporting that these two dimers could coexist within the same lattice structure. Importantly, most of the monomers inhabited orientations that aligned with the dimer populations, suggesting that monomers likely associate with the hydrophobic phase and then laterally assemble into a lattice (Figure 6). Finally, this shows that hydrophobic-to-hydrophilic cap mutations that weaken protein adsorption (11) can additionally interfere with film formation by altering the orientation of individual BslA monomers at the interface so that it is not consistent with the orientation of the monomers within the 2D lattice (12).

Our results support that the dimer1 and dimer2 interfaces are necessary for elastic film formation *in vitro* (Figure 4E) but the *in vivo* effects were less evident (Figure 4F) and amino acid substitutions in the dimer1 interface did not impact colony biofilm architecture. In the MD simulations, the binding is weakened, but both BslA^D1-^ and BslA^D2-^ dimers are still stable for about 10 ns before dissociation occurs. These data suggest that the film could in principle still form if stabilized by the remaining unaffected lateral interactions. Based on this evidence, the D1- and D2- mutants could form weak, but partially ordered 2d films. This is consistent with previous work wherein BslA mutations that weaken the elastic film could still form regular 2D films according to TEM images, although the size of the regular 2D domains was significantly decreased compared to WT BslA (11). The low *in vivo* impact of the dimer mutations may be due to the biofilm BslA films being stabilised by other factors such as interactions with other matrix components, making the effects of the dimer interface mutations muted when in the context of the whole biofilm. For instance, it has been postulated that the matrix protein TasA may interact with BslA (26). Whether these interactions are direct or specific has not been explored. To this end, our model of the BslA lattice may aid in future work as interaction in a mature biofilm matrix could occur between the assembled BslA film and its partners.

The limited *in vivo* effects led us to seek further validation of our findings using YweA. It was previously shown that YweA could form films *in vitro* but that the overexpression of the gene could not complement the absence of BslA *in vivo*. It had been hypothesised that this was due to the differences in film stability between the paralogues as well as the differences in the C-termini. Herein we show that YweA can rescue the △*bslA* strain when engineered to have the C-terminal region of BslA complemented by enhanced lateral interactions. These results further support that the film strength is important for BslA function in the biofilm. The increase in film strength seen with the YweA^D1+^ variant is congruent with the conserved packing structure in the lattice between paralogues.

The question remains of what the role is of secreted YweA? One study linked both *bslA* and *yweA* to sporulation repression in planktonic stationary conditions (27). This was attributed to the Spo0A pathway where YweA was shown to directly inhibit KinA autophosphorylation *in vitro*. Since KinA autophosphorylation occurs in the cytoplasm, it is possible that secreted YweA may play an additional, alternative role. In our current study, △*bslA* has the opposite effect with a huge decrease in sporulation rather than de-repression. These differences may be down to growth conditions or strain variation between PY79 (27) and NCIB 3610 which was used in this study. To our knowledge there is no literature on the role of secreted YweA besides the slight change to colony morphology seen between △*bslA* and the △*bslA*△*yweA* strain, which indicates that YweA has a small additive effect to BslA in the biofilm matrix (14).

The difference in colony biofilm architecture seen between the YweA^D1+^ vs YweA^CxC^ and YweA^D1+ CxC^ shows that the C-terminus of BslA has a unique role in BslA function outside of monolayer formation. This tail is essential for colony hydrophobicity but not film strength which is determined by the lateral interactions identified in this study. Previous investigation showed that covalently dimerized BslA (BslA^C-C^BslA) is associated at the interface using only one cap region of the two (13). Based on this, lateral interactions between BslA^C-C^BslA dimers could lead to a BslA bilayer in which one monomer of each pair is associated with the hydrophobic phase. The covalently bound monomers that remain in the hydrophilic phase could laterally interact since they are brought into proximity by their dimer partner. Whether this occurs, and leads to colony hydrophobicity, would need further investigation and is outside the limits of this study.

### Outlook

Film-forming proteins have been explored for their biotechnological uses. Fungal hydrophobins are arguably the class of surfactant proteins whose self-assembly behaviour at interfaces and potential in nanotechnology is most well characterized. For example, hydrophobin HFBII has been used to stabilize hydrophobic nanoparticles for efficient drug delivery (28). Furthermore, the molecular details of the hexagonal lattice formed by HFBI at interfaces (29) have been exploited to modulate protein interfacial properties such as film elasticity (30). A different system, bacterial S-layer proteins, has been used as a basis for isoporous filtration membranes and ordered films functionalized with molecules and nanoparticles (31). However, of the many proteins shown to form films, BslA is unique in the use of its cap conformational change to remain stable in solution while allowing strong interfacial association (Figure 6). Such conformational change orients the adsorbed protein (12) to facilitate lateral interactions, highlighting an exceptional degree of adaptability to remodelling. Understanding the determinates of BslA film formation may lead to new avenues of protein engineering, through an educated design of film modulation or exploitation such as particle display (15).

## Materials and Methods

### General growth conditions and strain construction

The *Bacillus subtilis* and *Escherichia coli* strains used and constructed in this study are detailed in Table S2. *E. coli* strain MC1061 was used for the construction and maintenance of plasmids. *B. subtilis* 168 derivatives were obtained by the transformation of competent cells with plasmids using standard protocols (32). SPP1 phage transductions were used to introduce DNA into *B. subtilis* strain NCIB 3610 (33). Both *E. coli* and *B. subtilis* strains were routinely grown in Lysogeny-Broth (LB) medium (10 g NaCl, 5 g yeast extract, and 10 g tryptone per litre) at 37°C for 16 hours. For complex colony formation, *B. subtilis* strains were grown on MSgg medium (5 mM potassium phosphate and 100 mM MOPS at pH 7.0 supplemented with 2 mM MgCl_2_, 700 μM CaCl_2_, 50 μM MnCl_2_, 50 μM FeCl_3_, 1 μM ZnCl_2_, 2 μM thiamine, 0.5% (vol/vol) glycerol, 0.5% (wt/vol) glutamate) solidified with 1.5% (wt/vol) Select Agar (Invitrogen) at 30°C for 48 hours (21) and images of colony biofilms were recorded using a Leica MZ16FA stereoscope as described previously (33). Ectopic gene expression was induced by medium supplementation with 25 μM isopropyl β-D-1-thiogalactopyranoside (IPTG) as indicated. When appropriate, antibiotics were used at the following concentrations: ampicillin 100 μg ml^-1^, chloramphenicol 5 μg ml^-1^, kanamycin 25 μg ml^-1^, and spectinomycin 100 μg ml^-1^.

### Plasmid construction and site-directed mutagenesis

All plasmids and primers used in this study and presented in Tables S3 and S4 and were constructed using standard methods. The plasmids for BslA_42-181_ derivatives overproduction were obtained by site-directed mutagenesis using the plasmid pNW1128 as template, which is a pGEX-6P-1 derivative used previously to overexpress BslA_42-181_ (34). Primers for the codon substitutions are included in Table S4 and mutagenesis was achieved following Stratagene Quikchange kit recommendations. The plasmids for overexpression *yweA_31-155_* or *yweA_31-155_* *E36F, T43K, V150D, D152A* (*yweA^D1+^*) were obtained using standard techniques with either NCIB 3610 or a synthetic DNA construct as the template DNA during PCR (see SI materials).

### Protein Purification

Proteins expressed and purified as previously published (10) and details can be found in the SI materials.

### Immunoblot Analysis

*B. subtilis* 48-h grown colony biofilms were collected from the agar plate by using a sterile loop and suspended in 250 μL of BugBuster Master Mix (Novagen) and the biomass was disrupted by passage through a 23 × 1 needle 10 times followed by gentle sonication to promote the release of the proteins from the biofilm matrix prior to immunoblot analysis. Further details are given in the SI materials.

### Biofilm hydrophobicity contact angle measurements

Biofilm hydrophobicity was evaluated by measuring the contact angle of a 5 μl droplet of water placed on the upper surface of the biofilm that had been grown for 48 hours at 30°C. Measurements were obtained using a ThetaLite TL100 optical tensiometer (Biolin Scientific) and analysed with OneAttension. The water droplet was allowed to equilibrate for 5 minutes prior imaging and measurement. Contact angles are present as the average of at least 3 independent experiments and the standard error of the mean associated with these values.

### Crystallisation, data collection, and processing

Purified YweA was methylated using the JBS Methylation Kit (Jena Bioscience), further purified using SEC, and concentrated to 15 mg/mL. Crystallisation screens including PEG/ion (Hampton Research), Crystal Screen 1+2 (Hampton Research), and JCSG-plus (Molecular Dimensions) were performed using sitting-drop vapour diffusion in 96 well plates at 20 °C. Drops consisted of 1:1 ratio YweA solution to mother liquor. Crystals formed in multiple conditions across the screens. A crystal that formed in condition 27 of the PEG/Ion screen (0.2 M sodium acetate trihydrate pH 8.0, 20% (wt/vol) polyethylene glycol) was cryo-protected transiently in mother liquor containing 15% (vol/vol) glycerol prior to vitrification with liquid nitrogen. Data were collected at a wavelength of 0.93 Å on the ID23-1 beamline at the European Synchrotron Radiation Facility (ESRF) in Grenoble. Images were collected using a Dectris Pilatus 2M detector and the data were processed with xia2 (35). The structure was solved by molecular replacement using MOLREP (36, 37) with the BslA structure (PDB ID4BHU) (10) as the search model. Molecular replacement was followed by iterative cycles of manual model building in Coot (38) and structure refinement by REFMAC5 (39, 40). The refined model statistics are shown in Table S1. The atomic coordinate has been deposited with RCSB PDB with accession code 5MKD. See supplemental method for details on structural alignment, analysis, and image creation.

### Sequence alignment

Sequences of BslA and YweA proteins from the *Bacillus* genus were obtained from the NCBI database and aligned using Clustal Omega (1.2.4) (41). The BslA family sequences used were *B. subtilis* BSLA_BACSU, *B. tequilensis* WP_174228711.1, *B. licheniformis* NVB34876.1, *B. amyloliquefaciens* UBZ24287.1, *B. cereus* CUB26733.1, *B. pumilus* WP_144533453.1. The YweA family sequence were B. subtilis A0A6M3ZHE7, *B. tequilensis* WP_024713112.1, *B. licheniformis* A0A1Q9FMA9_BACLI, *B. amyloliquefaciens* MCB5333331.1, *B. cereus* CUB21280.1, *B. mojavensis* A0A6H2JW44_BACMO, and *Laceyella tengchongensis* WP_154986593.1. The alignment was coloured and visualised using JalVeiw (42).

### Circular dichroism spectroscopy

Circular dichroism spectropolarimetry (CD) was performed by the Glasgow Structural Biology Biophysical Characterisation Facility. For further details see the SI materials.

### Wrinkle relaxation

Wrinkle relaxation data was collected using the Krüss EasyDrop DSA25 tensiometer. Proteins were diluted to 0.2 mg/ml in 25 mM phosphate buffer and loaded into a glass syringe with a needle diameter of 1.83 nm. A 40 *μ*l droplet of protein solution was expelled into glyceryl trioctanoate oil and allowed to equilibrate for 20 minutes at room temperature (10). Subsequently, the drop was compressed by withdrawing 10 *μ*l, thus inducing wrinkling of the surface film. For further details see the SI materials.

### TEM analysis

From the previously published TEM image of the BslA lattice (11), we obtained the average density within a unit cell from the inverse Fourier transform of the reciprocal lattice (so the 2D crystal Fourier transform) by selecting only the peaks corresponding to the periodicities of 3.9 nm and 4.3 nm (43). The resulting density within a 2x2 unit cell patch (each containing 2x2x2=8 proteins) was compared to what expected from our model by projecting the positions of the BslA octamer heavy atoms along the direction perpendicular to the interface using a Gaussian kernel with a standard deviation of 4 Å. We note that in our work we always represent BslA with the hydrophobic cap facing up, but in the TEM a BslA film is absorbed on a solid surface with the caps facing down, so to compare the experimental data with our model we considered the mirror image of the average unit cell density.

### Sporulation Assay

For heat-resistant spore quantification, colony biofilms were grown for 48 h at 30 °C. Cells were collected in 1 mL of saline solution, disrupted by passage through a 23 × 1 needle 10 times, and subsequently subjected to mild sonication (20% amplitude, 1 s on, 1 s off, for 5 s total) to liberate bacterial cells from the matrix. To kill vegetative cells, the samples were incubated for 20 min at 80 °C. To determine viable cell counts, serial dilutions were plated before and after the 80 °C incubation on LB agar supplemented with 100 μg·ml^-1^ spectinomycin and 5 μg·ml^-1^chloramphenicol for △*bslA*. The percentage of spores was established by colony-forming unit counting, and results are presented as the percentage of colony forming units obtained after incubation of the samples for 20 min at 80 °C, divided by the number of colony-forming units obtained before the heat inactivation.

## Supplementary Material

Supplemental Material

## Data Availability

All data for this study are included in the paper and/or within the supporting materials and at this repository (https://zenodo.org/record/8280824). The structure of YweA has been deposited to the PDB with accession code 5MKD.

## Significance

*Bacillus subtilis*, a common soil bacterium, forms water-repellent multicellular biofilm communities. A key component of this hydrophobicity is the presence of a self-assembled surface layer of protein known as BslA. Previous work has shown that BslA forms a highly ordered elastic layer at an interface, but the molecular interactions that drive self-assembly and film formation were not understood. In this work, we identify the key protein-protein interactions that facilitate film assembly using both experiment and simulation. The insights gained from this work will inform new engineering and design principles to control the biophysical properties of protein surface layers which has the potential to impact technologies reliant on interfacial stability such as emulsions, foams, and surface coatings.
