# Supplementary material for: Lateral interactions govern self-assembly of the bacterial biofilm matrix protein BslA
Source: Proc Natl Acad Sci U S A. Author manuscript; Available in PMC 2023 Nov 7. (PMC7615278; doi:10.1073/pnas.2312022120)
Supplement: Supplemental Material [file EMS190341-supplement-Supplemental_Material.pdf]

Supplemental Material

Lateral interactions govern self-assembly of the bacterial biofilm matrix protein BslA

Sofia Arnaouteli[%,1], Natalie C Bamford[%,1], Giovanni B Brandani[%,2], Ryan J Morris[3], Marieke Schor[4], Jamie T Carrington[5], Laura Hobley[6], Daan M F van Aalten[*,1,7], Nicola R Stanley-Wall[*,1], Cait E MacPhee[*,3]

[%] These authors contributed equally to this work and are listed alphabetically.
[1] Division of Molecular Microbiology, School of Life Sciences, University of Dundee, Dundee, DD5 4EH, UK
[2] Department of Biophysics, Graduate School of Science, Kyoto University, Japan
[3] National Biofilms Innovation Centre, School of Physics & Astronomy, University of Edinburgh, EH9 3FD Edinburgh, UK
[4] UB Education, Content & Support, Maastricht University
[5] Sir William Dunn School of Pathology, University of Oxford, South Parks Road, Oxford, OX1 3RE, UK
[6] School of Biosciences, University of Nottingham, Nottingham, UK
[7] Department of Molecular Biology and Genetics, University of Aarhus, Aarhus 8000, Denmark
[*] These authors share corresponding author status
Contact Prof Nicola Stanley-Wall for matters relating to molecular microbiology n.r.stanleywall@dundee.ac.uk
Contact Prof Cait MacPhee for matters relating to molecular dynamics and protein biophysics cait.macphee@ed.ac.uk

Contact Prof Daan van Aalten for matters relating to structural biology daan@mbg.au.dk

CRediT

Term	Definition	Initials
Conceptualization	Ideas; formulation or evolution of overarching research goals and aims	GBB DvA SA MS
Methodology	Development or design of methodology; creation of models	GBB SA NSW MS
Software	Programming, software development; designing computer programs; implementation of the computer code and supporting algorithms; testing of existing code components	GBB
Validation	Verification, whether as a part of the activity or separate, of the overall replication/ reproducibility of results/experiments and other research outputs	RJM
Formal analysis	Application of statistical, mathematical, computational, or other formal techniques to analyze or synthesize study data	GBB RJM DvA NSW SA NCB MS
Investigation	Conducting a research and investigation process, specifically performing the experiments, or data/evidence collection	GBB RJM NCB SA DvA LH
Resources	Provision of study materials, reagents, materials, patients, laboratory samples, animals, instrumentation, computing resources, or other analysis tools	SA
Data Curation	Management activities to annotate (produce metadata), scrub data and maintain research data (including software code, where it is necessary for interpreting the data itself) for initial use and later reuse	GBB NCB RJM
Writing - Original Draft	Preparation, creation and/or presentation of the published work, specifically writing the initial draft (including substantive translation)	RJM GBB NCB SA NSW CEM
Writing - Review & Editing	Preparation, creation and/or presentation of the published work by those from the original research group, specifically critical review, commentary or revision – including pre-or postpublication stages	GBB RJM NCB SA DvA NSW MS CEM
Visualization	Preparation, creation and/or presentation of the published work, specifically visualization/ data presentation	RJM NCB SA
Supervision	Oversight and leadership responsibility for the research activity planning and execution, including mentorship external to the core team	DvA NSW LH CEM
Project administration	Management and coordination responsibility for the research activity planning and execution	NSW CEM
Funding acquisition	Acquisition of the financial support for the project leading to this publication	NSW CEM

Supplemental Methods

Synthetic gene sequence

The sequence of the synthetic construct generated by Genescript was as follows: 5'-GCATCAATC**TTC**GCAAAAACAGTTAACAGC**AAG**AAAGAGTGGACCATTTCTGATATTGAAGTGACATATAAACCAAATGCGGTGCTTTCTCTTGGAGCGGTAGAATTTCAATTTCCTGACGGGTTTCATGCTACGACAAGAGATTCAGTGAATGGAAGAACACTGAAAGAAACACAGATTTTAAACGATGGAAAAACAGTCAGACTCCCGCTTACGCTTGATTTGTTAGGCGCATCCGAATTTGACCTTGTCATGGTGCGTAAAACTCTTCCTCGCGCAGGCACTTACACGATTAAAGGCGATGTAGTAAACGGTTTGGGAATCGGCAGTTTTTATGCTGAAACGCAGCTG**GAT**ATT**GCA**CCCCGTAGCACTCCTCCGACTCAGCCTTGCGGTTGCAACTAA -3'. The sequences in bold letters represent the Glu36Phe, The43Lys, Val150Asp and Asp152Ala point mutations and the underlined sequence represents the C-terminal *bslA* coding region and finally, the sequence in the upper case represents the mature region of the *yweA* gene sequence.

Immunoblot analysis

The samples released from the biofilm matrix were incubated at room temperature with agitation for 20 min, and the insoluble cell debris was removed by centrifugation at 17,000 × g for 10 min at 4°C. 1.5 µg of total protein extract was separated on a 14% (wt/vol) SDS/PAGE before transfer onto PVDF membrane (Millipore) by electroblotting at 25 V for 2 h. The membrane was incubated for 16 h in 3% (wt/vol) powdered milk in TBS [20 mM Tris·HCl (pH 8.0) and 0.15 M NaCl] at 4°C with shaking. This step was followed by 2-h incubation with purified anti-BslA antibody at a dilution of 1:500 (vol/vol) in TBS in 3% (wt/vol) powdered milk wash buffer (TBS + 0.05% (vol/vol) Tween 20). The membrane was washed by using wash buffer (TBS + 0.05% (vol/vol) Tween 20) and incubated for 45 min with the secondary antibody

conjugated to horseradish peroxidize [goat anti-rabbit (Pierce)] at a dilution of 1:5,000 (vol/vol). The membrane was washed, developed, and visualised with the Azure 600 Imager. Western blot analysis for YweA detection was performed as described above with purified anti-YweA antibody at a dilution of 1:10,000 (vol/vol).

Protein Purification.

BslA$_{42\text{-}181}$ protein and its derivatives, BslA$_{42\text{-}181\ D66K\ N101D}$ and BslA$_{42\text{-}181\ F51A\ D166K}$ were overexpressed and purified as previously described (1). Briefly, the pGEX-1-6P derivative plasmids were introduced into *E. coli* BL21 (DE3). After growth and overexpression, the protein was purified in HEPES buffer. To achieve this, the *E. coli* cells were lysed in an Emulsiflex cell disruptor and the solubilized protein extracts, cleared of cell debris, were incubated with Glutathione Sepharose 4B (GE Healthcare), allowing the fused protein to bind to the GST binding beads. After incubation, the beads containing the BslA-fusion were recovered using a gravity flow column (Bio-Rad) and suspended into new buffer containing DTT and TEV-His tagged protease. TEV removes the GST tag from BslA protein, which remains soluble in the purification buffer. The protease and unbound GST are then separated from BslA by incubating the mixture with Ni-nitrilotriacetic acid agarose (Qiagen) and Glutathione beads, followed by a new passage in a gravity flow column. The flow-through recovered contains the purified protein, which is then concentrated using VivaSpin concentrator.

To purify YweA$_{31\text{-}155}$ and its derivative YweA$_{33\text{-}155\ E36F,\ T43K,\ V150D,\ D152A}$ and the required strains containing the expression plasmids were grown and the protein was overexpressed and purified as previously described (1). Briefly, the required coding regions were introduced into pET-15b-TEV vector (see Tables S3 and S4). The pET-15b-TEV derivative plasmids were introduced into *E. coli* BL21 (DE3). After growth and overexpression *E. coli* cells were

suspended in HEPES buffer (300 mM NaCl, 50 mM HEPES pH8 with the addition of 20 mM imidazole) containing protease inhibitors (Roche c*O*mplete EDTA-free) and lysed using the Emulsiflex C3. The solubilized protein extracts, cleared of cell debris, were incubated with Ni-nitrilotriacetic acid agarose (Qiagen) for 4 hours, allowing the fused protein to bind to the Ni-NTA binding beads. After incubation, the beads containing the YweA-fusion proteins were recovered using a gravity flow column (Bio-Rad) and suspended into new buffer (300 mM NaCl, 50 mM HEPES pH 8.0 with the addition of 250 mM imidazole) on column for half an hour to promote the protein release. Proteins were recovered by collection of the column flow-through which was incubated overnight in buffer 300 mM NaCl, 50 mM HEPES pH 8.0 with the addition of DTT and TEV-His tagged protease. VivaSpin concentrators were used to remove the imidazole in solution by replacement up with the purification buffer. The protein recovered from the concentrators was incubated overnight with Ni-NTA beads to bind to the His-tag fragments released, followed by a new passage in a gravity flow column. The flow-through recovered contains the purified protein, which is then concentrated using VivaSpin concentrators. The resultant protein samples were analysed by using 14% (wt/vol) SDS-PAGE, with and without the addition of a reducing agent (β-mercaptoethanol) in the loading dye and stained with Instant Blue before photography.

Wrinkle Relaxation Measurements

The evolution of the wrinkles was captured by a CCD camera for up to 10 minutes. ImageJ was used for image analysis. A line profile was drawn across the wrinkles and plotted using greyscale values (0-255) of all pixels along this line. 5 to 10 wrinkles were measured on each droplet, with a total of 3 independent droplets measured for each protein. To plot the relaxation rate, the greyscale values were background corrected and normalised.

Chircular dichroism spectroscopy

Assays were performed using a Jasco J-810 spectropolarimeter (serial number B029360750) at concentrations of between 0.35 - 0.5 mg/mL in a 0.02 cm quartz cuvette. Scans were performed in continuous mode between 260-190 nm at a spread of 10 nm/min and a band width of 1 nm. The data pitch was 0.2 nm with response time of 2 s. Three scans were accumulated and averaged to produce the final curve.

Structure alignment and analysis.

Structural alignments were performed using Coot (version 0.8.9.2)(2). To compare the four monomers in the asymmetric unit alignment was calculated using LSQ superimposition (least-squares method) over all α-carbons and over all atoms using Chain B as reference. Comparison of BslA (PDB 4HBU chain C) to YweA (chain B) was done using secondary structure comparison with the SSM superpose tool in Coot. Coordinates of aligned structures were visualised in PyMOL (version 2.5.2, © Schrodinger, LLC) after running *Dss* (define secondary structure) on all structures for consistency. All images of protein structures were produced using PyMOL. Visualisation of YweA hydrophobic regions was done using the color_h.py script (https://pymolwiki.org/index.php/Color_h). Electrostatics of the YweA structure were calculated using default setting in the PyMOL APBS GUI Plugin (3).

MD simulations details

All-atom MD simulations were performed using the GROMACS 4 (4) software package. The protein was represented according to the AMBER ff99SB*-ILDN force field (5-7) and the TIP3P model was used to represent the water molecules(8). All bonds were constrained to their equilibrium value using the LINCS algorithm(9), whereas the geometry of the water molecules was constrained using the SETTLE algorithm(10). Van der Waals and short-range Coulomb

interactions were both cut-off at 10 Å, whereas long-range electrostatics was treated using the particle mesh Ewald method(11) with a Fourier spacing of 1.2 Å. The equations of motion were integrated using the leap-frog algorithm (12) with a time-step of 2 fs. In all cases, the production simulation runs were performed in the NVT ensemble, using the Nose-Hoover thermostat(13) with a reference temperature of 300 K and a relaxation time of 1 ps; solvent and solute were thermostated independently. The size of the simulation box is set so that the protein is always at least 12 Å away from the closest image of the box. The net charge of the protein was neutralised via the addition of a minimum amount of either Na or Cl ions (depending on the net charge of the BslA mutant). Production runs are preceded by an equilibration protocol consisting of an energy minimisation with the steepest descent algorithm and a 200 ps run in the NVT ensemble with the protein's heavy atoms restrained to their initial positions by a harmonic potential with spring constant of 1000 kJ/mol/nm^2.

The first dimer (dimer1) consists of chains C and H of the BslA crystal structure (1). To create the second dimer (dimer2), chain C of the BslA crystal structure was aligned to chains A and B of the YweA crystal structure using PyMOL. Note that a truncated version of BslA, comprising residues 48 to 170 was used for all simulations. PyMOL was used to generate point mutations Phe51Ala, Asp66Lys, Asn101Asp, Asp166Lys to create the Phe51Ala-Asp166Lys and Asp66Lys-Asn101Asp double mutants.

The stability of two distinct BslA dimers adsorbed to an air-water interface was characterised using both equilibrium and steered MD simulations. For the unbiased simulations, the dimers are placed in a rectangular box with sides 88x85x105 ˚A and 94x94x105 ˚A for dimer1 and dimer 2 respectively, and they were solvated with water (which occupies 65% of the volume) so that the two proteins are already adsorbed at the air/water

interface. The system is then run in the NVT ensemble for 100 ns unless a complete dissociation of the two monomers occurs (which occurs for mutants). For the unbiased simulations of the individual monomers, the protein is initially placed at the centre of the water phase of the system and then allowed to spontaneously adsorb to the air interface. The distribution of interfacial orientations has been computed from 16 independent simulation runs based on the configurations adopted after adsorption takes place in each run (overall, we produced ~1.7 µs of total simulation time in the adsorbed state).

The potential of mean force (PMF) was calculated from pulling MD simulations based on the Jarzynski equality (14). For these simulations, the dimers are adsorbed at an air interface within a 90x77.6x105 Å simulation box, aligned along the x direction. During pulling, one monomer acts as a reference, with its backbone Cα atoms restrained at their initial positions by a harmonic spring constant of 1000 $kJ/mol/nm^2$, while the other is pulled along x at a speed of 0.2 Å/ns, using a harmonic potential with a spring constant of 10000 $kJ/mol/nm^2$. Each of the two PMF profiles have been obtained from 32 independent pulling MD runs using the cumulant expansion of the Jarzynski equality, since this is regarded as being more robust than the Jarzynski equality itself(14). The errors in the potentials of mean force have been calculated using the Jackknife algorithm(15). The standard free energy of binding of the dimers have been estimated from the PMF using $\Delta G_{bind} = k_B T \ln(K_{eq} C^0)$ (16), with the thermal energy $k_B T$=2.5 kJ/mol, C^0=1/1661 $Å^3$, and the equilibrium constant $K_{eq} = 4\pi r_{bulk}^2 \int_{site} dr\, e^{-\beta[W(r)-W(r_{bulk})]}$ is a distance where the two monomers do not interact (we choose 28 Å for dimer1 and 33 Å for dimer2), the integral runs over the binding site, and $W(r)$ is the distance-dependent PMF from the steered MD simulations.

Table S1. Crystallography data collection and refinement statistics.

**Data collection**	
Resolution (Å)	50-2.51 (2.6-2.51)[a]
Space group	*C222*
Unit cell (Å)	121.4 128.3 84.3
Unit cell angles (°)	90 90 90
Observed reflections	118718 (11356) [a]
Mean *I*/s (*I*)	16.1 (1.6) [a]
Completeness (%)	99.5 (65.2) [a]
Multiplicity	5.2 (5.2) [a]
$CC_{(1/2)}$	0.99 (0.65) [a]
R_{merge} (%)	6.7 (92) [a]
**Refinement**	
R_{factor} / R_{free} (5%)	0.208 (0.263)
Protein residues	495
Solvent Molecules	23
Average *B*-factor (Å^2)	56.6
RMSD (bonds, Å)	0.012
RMSD (angle, °)	1.6

[a] The number in brackets represent data from the highest resolution shell.

Table S2 Full list of strains used in this study.

Strain	Relevant genotype /Description [a]	Source / Construction [b,c]
Bacillus subtilis		
NCIB 3610	Prototroph	BGSC
168	*trpC2*	BGSC
NRS2097	NCIB 3610 *bslA::cml*	(17)
NRS2299	NCIB 3610 *bslA:: cml amyE::Phy-spank-bslA-lacI (spc)*	(17)
NRS5519	168 *amyE::Phy-spank-bslA*$_{D66KN101K}$*-lacI (spc)*	pNW1475 → 168
NRS5521	168 *amyE::Phy-spank-bslA*$_{F51AD166K}$*-lacI (spc)*	pNW1709 → 168
NRS5524	NCIB 3610 *bslA::cml amyE::Phy-spank-bslA*$_{D66KN101K}$*-lacI (spc)*	SPP1 NRS5519 → NRS2097
NRS5526	NCIB 3610 *bslA:: cml amyE::Phy-spank-bslA* $_{F51A\ D166K}$*-lacI (spc)*	SPP1 NRS5521 → NRS2097
NRS2405	NCIB 3610 *yweA::kan*	(18)
NRS2412	NCIB 3610 *bslA::cml amyE::Phy-spank- yweA-lacI (spc)*	(19)
NRS4834	NCIB3610 *bslA::cml amyE::Phy-spank-bslAss-yweA-* $_{bslA\ 171\text{-}181}$ *-lacI (spc)* termed *yweA*^{CxC}	(18)
NRS5540	168 *amyE::Phy-spank-bslAss-yweA*$_{E36F,\ T43K,\ V150D,\ D152A}$ *-lacI (spc)*	pNW1715 → 168
NRS5539	168 *amyE::Phy-spank- bslAss-yweA*$_{E36F,\ T43K,\ V150D,\ D152A)\text{-}bslA\ 171\text{-}181}$ *-lacI (spc)* termed *yweA*$^{D1+}$	pNW1714 → 168
NRS5542	NCIB 3610 *bslA:: cml amyE::Phy-spank- bslAss-yweA*$_{E36F,\ T43K,\ V150D,\ D152A}$ *-lacI (spc)*	SPP1 NRS5540 → NRS2097
NRS5541	NCIB3610 *bslA::cml amyE::Phy-spank- bslAss-yweA*$_{E36F,\ T43K,\ V150D,\ D152A)\text{-}bslA\ 171\text{-}181}$ *-lacI (spc))* termed *yweA*$^{D1+CxC}$	SPP1 NRS5539 → NRS2097
NRS5551	NCIB 3610 *bslA::cml amyE::Phy-spank-bslAss-yweA (spc)*	(18)
E. coli		
MC1061	*E. coli F'lacIQ lacZM15 Tn10* (*tet*)	*E. coli* Genetic Stock Centre
BL21 (DE3)	F– ***omp***T ***hsd***SB(rB–, mB–) ***gal dcm*** (DE3)	(20)

[a] Drug resistance cassettes are indicated as follows: *cml*, chloramphenicol resistance; kan, kanamycin resistance; *spc*, spectinomycin resistance.

[b] The direction of strain construction is indicated with DNA or phage (SPP1) (→) recipient strain.

[c] The citation is provided if the strain has previously been described. BSGC represents the *Bacillus* genetic stock centre.

Table S3. Plasmids used in this study

Plasmid	Description [a]	Source [b]
pDR111	*B. subtilis amyE* integration vector for IPTG-induced expression	(21)
pQE70	Cloning vector for His-tag fusions	Qiagen
pGEX-6P-1	Vector for production of GST-fused proteins	GE Healthcare
pET15b-TEV	Vector for production of His tag-fused proteins	Lab sources
pNW1420	pET15bTEV-*yweA*$_{31\text{-}155}$	(19)
pNW1473	pDR111-*bslA* $_{D66K}$	This work
pNW1475	pDR111-*bslA* $_{D66K\ N101D}$	This work
pNW1476	pDR111- *bslA* $_{F51A}$	This work
pNW1709	pDR111-*bslA* $_{F51A\ D166K}$	This work
pNW1128	pGEX-TEV-*bslA*$_{42\text{-}181}$	(1)
pNW1468	pGEX-TEV-*bslA* $_{D66K}$	This work
pNW1471	pGEX-TEV- *bslA*$_{D66K\ N101D}$	This work
pNW1467	pGEX-TEV-*bslA* $_{D166K}$	This work
pNW1472	pGEX-TEV- *bslA* $_{F51A\ D166K}$	This work
pNW611	pQE70-*bslA* signal sequence (hereafter *bslAss*)	(17)
pNW1713	pQE70- *bslAss-yweA* $_{E36F,\ T43K,\ V150D,\ D152A}$	This work
pNW1714	pDR111-*bslAss*- *yweA*$_{E36F,\ T43K,\ V150D,\ D152A\text{-}bslA\ 171\text{-}181}$	This work
pNW1715	pDR111-*bslAss*- *yweA*$_{E36F,\ T43K,\ V150D,\ D152A}$	This work
pNW1079	pDR111- *bslAss*- *yweA-bslA*$_{171\text{-}181}$	(18)
pNW1717	pET15b-TEV- yweA$_{E36F,\ T43K,\ V150D,\ D152A}$	This work
pNW512	pDR111-*bslA*	(22)
pNW1719	pDR111- *bslAss*- *yweA*	(18)

a. The genotype of the plasmid is described.

b. Relevant information for the construction of the plasmid is provided along with the references if previously published.

Table S4. Oligonucleotide primers used in this study.

Primer	Sequence 5' – 3' [a]	Use [b]
NSW12	CGATTCAAAACCTCTTTACTG	*amyE* locus for assessing double crossovers
NSW13	GCTTAAGCCCGAGTC	*amyE* locus for assessing double crossovers
NSW1853	GTACCATATGCAGTCTGCATCAATCGAG	*yweA*$_{31\text{-}155}$ cloning
NSW1854	GATCCTCGAGTTATTAACGGGGATCAATCAC	*yweA*$_{31\text{-}155}$ cloning
NSW1719	GTCTACAGCTTCATTG**gca**GCAACAATCACTGGCG	SDM on pNW512/pNW1128 to convert Phe51 to Ala
NSW1720	CGCCAGTGATTGTTGC**tgc**CAATGAAGCTGTAGAC	SDM on pNW512/pNW1128 to convert Phe51 to Ala
NSW2042	AGCCAGCATT**aaa**GTGGCTAAGC	SDM on pNW1476/pNW1466 to convert Asp166 to Lys
NSW2049	GCTTAGCCAC**ttt**AATGCTGGCT	SDM on pNW1476/pNW1466 to convert Asp166 to Lys
NSW2044	GTCTTTCTCA**aaa**ATCGAATTGACTTACC	SDM on pNW512/pNW1128 to convert Asp66 to Lys
NSW2045	CATTCCGTTTTGCTGGCG	SDM on pNW512/pNW1128 to convert Asp66 to Lys
NSW2046	ATTGAACGGA**gat**GCCTTGCGTA	SDM on pNW1473/pNW1468 to convert Asn101 to Asp
NSW2047	GTGTCTTTCGTGTTTGCAG	SDM on pNW1473/pNW1468 to convert Asn101 to Asp
NSW2050	ATGCA<u>AGATCT</u>GCATCAATCTTCGCAAAAACAG	Forward primer for cloning chimeric YweA with Glu36Phe, Thr43Lys, Val150Asp, Asp152Ala mutations into pNW611
NSW645	AACT<u>GCATGC</u>TTAGTTGCAACCGCAAGGCTGA	Reverse primer for cloning chimeric YweA with Glu36Phe, Thr43Lys, Val150Asp, Asp152Ala mutations into pNW611
NSW2051	AACT<u>GCATGC</u>TTAACGGGGTGCAATATCCAGCT	Reverse primer for cloning chimeric YweA without *bslA*$_{171\text{-}181}$ with Glu36Phe, Thr43Lys, Val150Asp, Asp152Ala mutations into pNW611
NSW2052	GTAC<u>CATATG</u>CAGTCTGCATCAATCTTC	Forward primer for cloning chimeric YweA with mutations Glu36Phe, Thr43Lys, Val150Asp, Asp152Ala into pET15b
NSW2053	ATAC<u>CTCGAG</u>TTAACGGGGTGCAATATCCA	Reverse primer for cloning chimeric YweA with mutations Glu36Phe, Thr43Lys, Val150Asp, Asp152Ala into pET15b
NSW1912	ATAC<u>CTCGAG</u>TTAGTTGCAACCGCAA	Reverse primer for cloning chimeric YweA without bslA$_{171\text{-}181}$ with mutations Glu36Phe, Thr43Lys, Val150Asp, Asp152Ala into pET15b

[a] Restriction sites are underlined and sites for mutations are highlighted in lower case bold.

[b] SDM = site directed mutagenesis and relevant information for the use of the primers is provided.

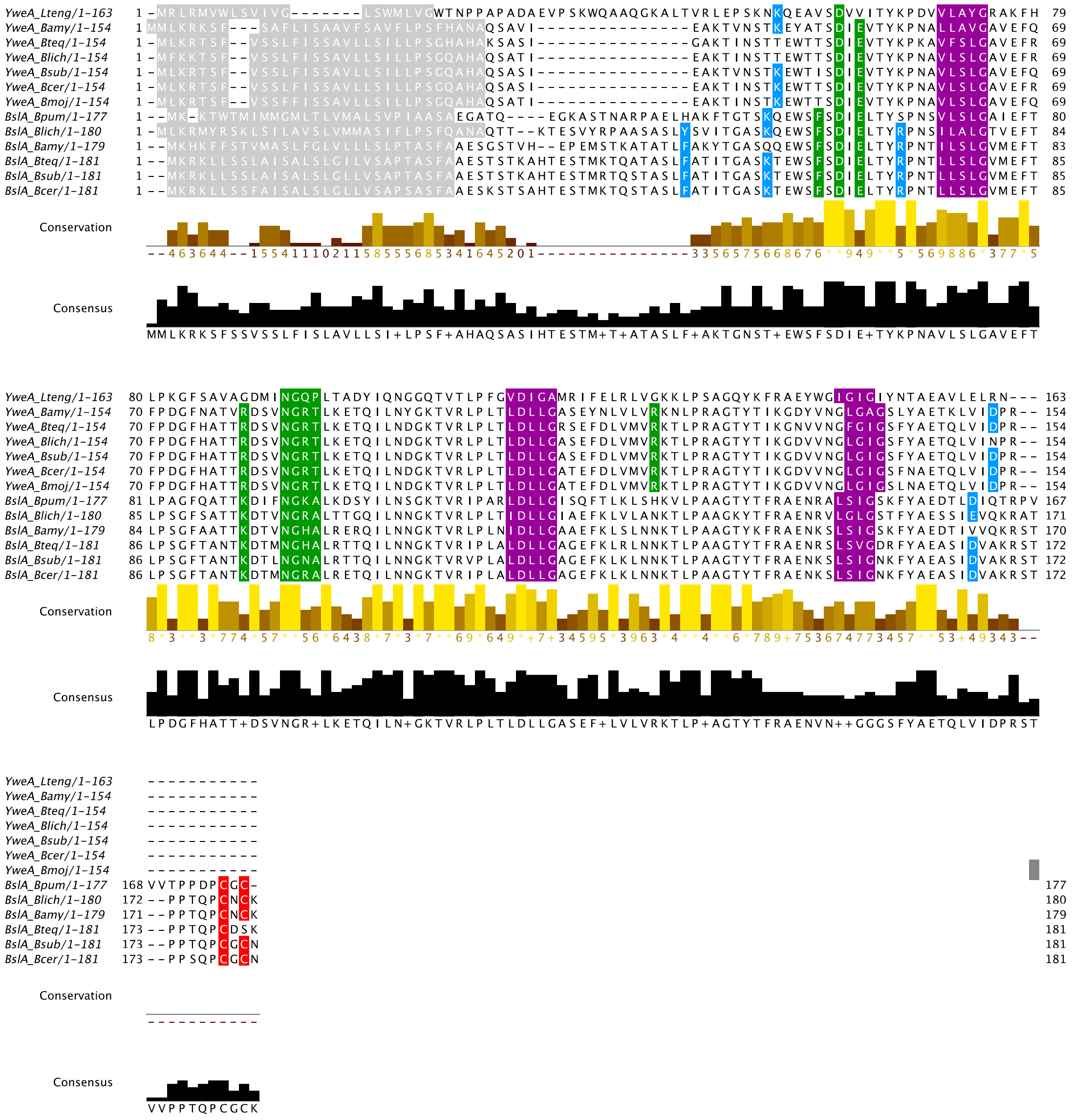

Figure S1. Sequence alignment of BslA and YweA protein sequences. Sequences include those listed in the method section and are labelled in the alignment with their species name shortened to the first letter of the genus followed directly by the first few letters of the species (i.e., *Bacillus subtilis* is "*Bsub*"). The predicted signal peptides are highlighted in grey. Residues known, or predicted, to be in the hydrophobic cap are coloured in purple. Residues of the Dimer1 interface are coloured blue and Dimer2 in green. The CxC motif is highlighted in red. Conservation and consensus graphs were calculated by JalView. Alignment was performed using ClustalW and visualised in JalView before figure creation in Adobe Illustrator.

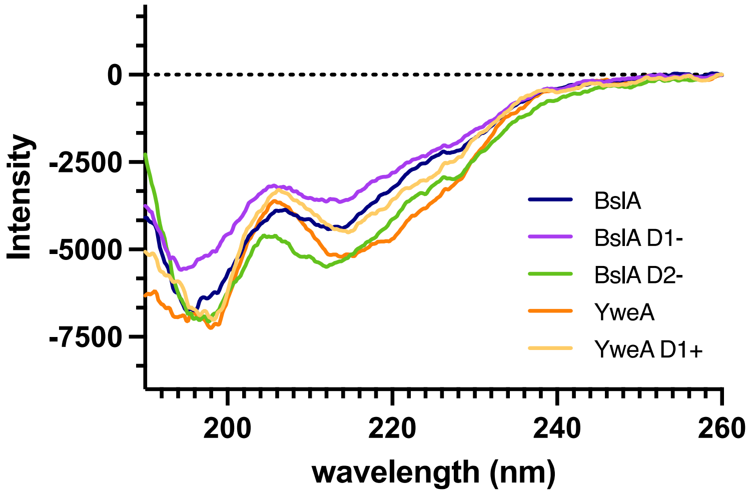

Figure S2. Representative CD spectra of BslA and YweA recombinant proteins.

Supplemental References

1. L. Hobley *et al.*, BslA is a self-assembling bacterial hydrophobin that coats the *Bacillus subtilis* biofilm. *Proceedings of the National Academy of Sciences of the United States of America* **110**, 13600-13605 (2013).
2. P. Emsley, K. Cowtan, Coot: model-building tools for molecular graphics. *Acta crystallographica. Section D, Biological crystallography* **60**, 2126-2132 (2004).
3. E. Jurrus *et al.*, Improvements to the APBS biomolecular solvation software suite. *Protein Sci* **27**, 112-128 (2018).
4. B. Hess, C. Kutzner, D. van der Spoel, E. Lindahl, GROMACS 4: Algorithms for highly efficient, load-balanced, and scalable molecular simulation. *Journal of Chemical Theory and Computation* **4**, 435-447 (2008).
5. R. B. Best, G. Hummer, Optimized Molecular Dynamics Force Fields Applied to the Helix-Coil Transition of Polypeptides. *J Phys Chem B* **113**, 9004-9015 (2009).
6. V. Hornak *et al.*, Comparison of multiple amber force fields and development of improved protein backbone parameters. *Proteins* **65**, 712-725 (2006).
7. K. Lindorff-Larsen *et al.*, Improved side-chain torsion potentials for the Amber ff99SB protein force field. *Proteins* **78**, 1950-1958 (2010).
8. W. L. Jorgensen, J. Chandrasekhar, J. D. Madura, R. W. Impey, M. L. Klein, Comparison of Simple Potential Functions for Simulating Liquid Water. *J Chem Phys* **79**, 926-935 (1983).
9. B. Hess, H. Bekker, H. J. C. Berendsen, J. G. E. M. Fraaije, LINCS: A linear constraint solver for molecular simulations. *J Comput Chem* **18**, 1463-1472 (1997).
10. S. Miyamoto, P. A. Kollman, Settle - an Analytical Version of the Shake and Rattle Algorithm for Rigid Water Models. *J Comput Chem* **13**, 952-962 (1992).
11. T. Darden, D. York, L. Pedersen, Particle mesh Ewald: An N·log(N) method for Ewald sums in large systems. *J Chem Phys* **98**, 10089–10092 (1993).
12. R. W. Hockney, S. P. Goel, J. W. Eastwood, Quiet High-Resolution Computer Models of a Plasma. *J Comput Phys* **14**, 148-158 (1974).
13. S. Nose, A Molecular-Dynamics Method for Simulations in the Canonical Ensemble. *Mol Phys* **52**, 255-268 (1984).
14. S. Park, K. Schulten, Calculating potentials of mean force from steered molecular dynamics simulations. *J Chem Phys* **120**, 5946-5961 (2004).
15. B. Efron *et al.*, *The jackknife, the bootstrap, and other resampling plans*, CBMS-NSF regional conference series in applied mathematics 38 (Society for Industrial and Applied Mathematics (SIAM, 3600 Market Street, Floor 6, Philadelphia, PA 19104), Philadelphia, Pa, 1982).
16. T. Siebenmorgen, M. Zacharias, Evaluation of Predicted Protein-Protein Complexes by Binding Free Energy Simulations. *J Chem Theory Comput* **15**, 2071-2086 (2019).
17. A. Ostrowski, A. Mehert, A. Prescott, T. B. Kiley, N. R. Stanley-Wall, YuaB functions synergistically with the exopolysaccharide and TasA amyloid fibers to allow biofilm formation by *Bacillus subtilis*. *J Bacteriol* **193**, 4821-4831 (2011).
18. S. Arnaouteli *et al.*, Bifunctionality of a biofilm matrix protein controlled by redox state. *Proc Natl Acad Sci U S A* **114**, E6184-E6191 (2017).
19. R. J. Morris *et al.*, Natural variations in the biofilm-associated protein BslA from the genus Bacillus. *Sci Rep* **7**, 6730 (2017).
20. F. W. Studier, B. A. Moffatt, Use of bacteriophage T7 RNA polymerase to direct selective high-level expression of cloned genes. *Journal of molecular biology* **189**, 113-130 (1986).
21. R. A. Britton *et al.*, Genome-wide analysis of the stationary-phase sigma factor (Sigma-H) regulon of *Bacillus subtilis*. *J Bacteriol* **184**, 4881-4890 (2002).
22. D. T. Verhamme, E. J. Murray, N. R. Stanley-Wall, DegU and Spo0A jointly control transcription of two loci required for complex colony development by Bacillus subtilis. *J Bacteriol* **191**, 100-108 (2009).
